# Cytological Classification Diagnosis for Thyroid Nodules via Multimodal Model Deep Learning

**DOI:** 10.1002/advs.202511369

**Published:** 2025-10-16

**Authors:** Yuanzheng Lou, Yongjian Su, Haoda Lu, Wencai Li, Weihua Yin, Shengnan Li, Huobiao Zhu, Kok Haur Ong, Gang Chen, Yong Jiang, Yifei Liu, Shenglei Li, Manchun Yang, Zhengyu Zhang, Xiaoyan Wang, Xiaohui Zhu, Xinmi Huo, Longjie Li, Chao Wang, Nanyan Zhang, Weijun Pan, Bin Shang, Xudan Yang, Yongqiang Zhu, Xiaolin Liu, Weimiao Yu, Xiuwu Bian, Yanqing Ding

**Affiliations:** ^1^ Department of Pathology Nanfang Hospital and School of Basic Medical Sciences Southern Medical University Guangzhou 510515 China; ^2^ College of Bioengineering Chongqing University Chongqing 400030 China; ^3^ Institute of Pathology & Southwest Cancer Center the First Affiliated Hospital (Southwest Hospital) and School of Basic Medical Sciences Army Medical University (Third Military Medical University), and the Key Laboratory of Tumor Immunopathology the Ministry of Education (Third Military Medical University) Chongqing 400038 China; ^4^ Chongqing Institute of Advanced Pathology Jinfeng Laboratory Chongqing 400041 China; ^5^ Guangzhou F.Q. PATHOTECH Co., Ltd Guangzhou 510515 China; ^6^ Guangzhou Huayin Health Medical Group Co., Ltd Guangzhou 510663 China; ^7^ Intelligent Digital and Molecular Pathology (IDMP) Lab Bioinformatics Institute (BII) Agency of Science Technology and Research (A*STAR) Singapore 138671 Singapore; ^8^ Department of Pathology The First Affiliated Hospital of Zhengzhou University No. 1 Jian She Dong Avenue Zhengzhou 450002 China; ^9^ Department of Pathology Peking University Shenzhen Hospital Shenzhen 518036 China; ^10^ Department of Pathology Clinical Oncology School of Fujian Medical University Fujian Cancer Hospital Fuzhou Fujian 35000 China; ^11^ Department of Pathology West China Hospital of Sichuan University Chengdu 610044 China; ^12^ Department of Pathology Affiliated Hospital of Nantong University and Medical School of Nantong University Nantong 226007 China; ^13^ Guangdong Province Key Laboratory of Molecular Tumor Pathology Guangzhou 510515 China; ^14^ Department of Pathology Sichuan Provincial People's Hospital School of Medicine University of Electronic Science and Technology of China Chengdu 610072 China; ^15^ Computational & Molecular Pathology Lab (CMPL) Institute of Molecule and Cell Biology (IMCB) Agency of Science Technology and Research (A*STAR) Singapore 138673 Singapore

**Keywords:** artificial intelligence, cytopathological diagnosis, fine needle aspiration cytology (FNAC), thyroid nodules, whole‐slide image (WSI)

## Abstract

The rising prevalence of thyroid nodules is straining limited cytopathology resources, resulting in excessive overdiagnosis and overtreatment with significant patient and healthcare consequences. To address this, AI‐TFNA is developed, a robust artificial intelligence platform leveraging extensive clinical data to enhance diagnostic accuracy and clinical efficiency. A total of 20,803 thyroid samples are collected from seven medical centers across different regions in China. Of these, 4,421 thyroid fine‐needle aspiration (TFNA) samples from three hospitals are used to train AI‐TFNA, ensuring strong generalizability across diverse clinical settings. For the internal validation, AI‐TFNA demonstrates exceptional performance: the overall accuracy of TBS I is 93.27%, the sensitivity of TBS V and TBS VI reaches 85.37% and 83.78%, while the specificity of TBS II is 97.13%. Consistent results are observed in an external cohort of 2,153 samples, demonstrating robust generalizability. The incorporation of BRAF mutation data into AI‐TFNA and the development of a multi‐modal model further improve precision by significantly improving the differentiation between benign and malignant thyroid nodules. Image Appearance Migration (IAM) is an innovative technique that substantially improves cross‐institutional model generalizability, increasing AI‐TFNA sensitivity by 1.90% and specificity by 8.12%. AI‐TFNA offers rapid, reliable decision support, advancing thyroid nodule diagnostics.

## Graphical abstract

This study introduces AI‐TFNA, an innovative artificial intelligence model designed to assist cytopathologists in classifying thyroid nodules based on The Bethesda System for Reporting Thyroid Cytopathology (TBSRTC). The model effectively differentiates between benign and malignant thyroid nodules, demonstrating significant potential as a screening tool to enhance diagnostic accuracy and workflow efficiency in clinical practice.

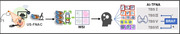

## 1 Introduction

**Figure 1:**
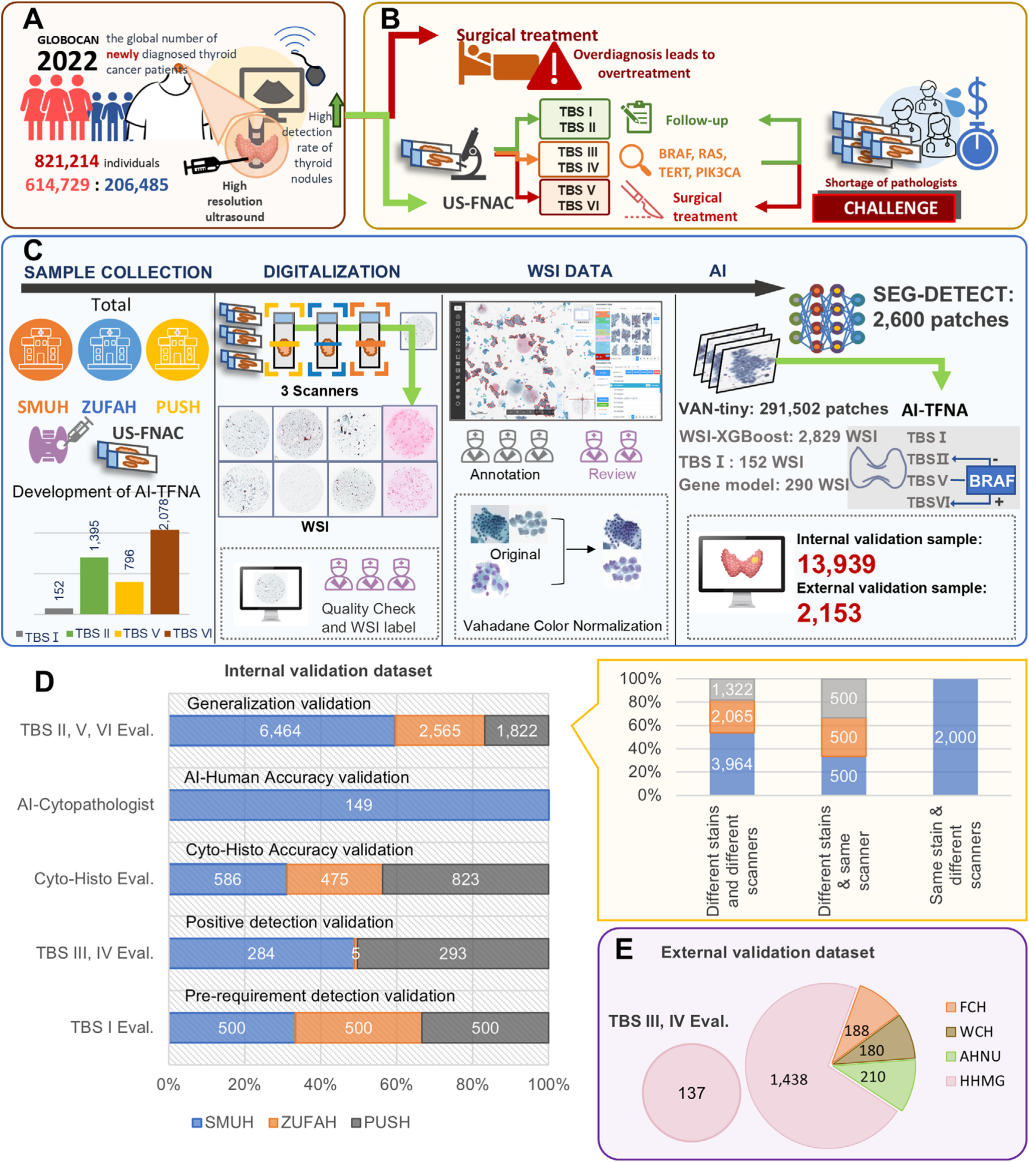
Study background and study profile. A, B). According to the GLOBOCAN 2022 database, the incidence of thyroid nodules is increasing annually. Clinically, thyroid nodules are initially evaluated using high‐resolution ultrasound. Suspected malignant nodules are then examined by ultrasound‐guided FNAC. Diagnosis and treatment follow the results provided by TBSRTC. Currently, the increasing incidence of thyroid nodules places considerable pressure on pathologists, leading to a high rate of overdiagnosis and overtreatment. This situation adversely impacts patients' physical and mental health and results in substantial time and financial losses. C). In order to solve this problem, we designed an AI model (AI‐TFNA) to assist pathologists in diagnosis. We collected thyroid fine needle aspiration cytology samples from SMUH, ZUFAH, and PUSH. These samples were scanned using three different scanners to obtain WSI. The training datasets for the deep learning models were created through manual annotation, semi‐supervised annotation, and data augmentation. Based on this dataset, we trained an integrated system consisting of four parts: SEG‐DETECT (nuclear segmentation), VAN‐tiny (cell classification), XGBoost (WSI‐level diagnosis), and a decision tree of TBSI. Finally, we evaluated the performance of the model on both internal and external samples. D). The composition of datasets in internal validation (SMUH, ZUFAH, and PUSH). E). The external validation datasets came from FCH, WCH, AHNU, and HHMG. FNAC: Fine Needle Aspiration Cytology; TBSRTC: The Bethesda System for Reporting Thyroid Cytopathology; SMUH: Southern Medical University's Nan Fang Hospital; ZUFAH: The Zhengzhou University First Affiliated Hospital; PUSH: Peking University Shenzhen Hospital; WSI: Whole Slide Images; FCH: Fujian Cancer Hospital; WCH: West China Hospital, Sichuan University; AHNU: Affiliated Hospital of Nantong University; HHMG: Huayin Health Medical Group.

Thyroid nodules are prevalent endocrine disorders, affecting over 60% of the general population, as shown in **Figure** **1**A, with a particularly significant occurrence among women.^[^ ^1^, ^2^ ^]^ While most of these nodules are benign and asymptomatic, malignant thyroid nodules comprise about 5%–17% of cases,^[^ ^3^, ^4^ ^]^ and the mortality rate associated with thyroid cancer has remained relatively stable.^[^ ^5^ ^]^ Data from the Global Cancer Observatory of the World Health Organization's International Agency for Research on Cancer in 2022 indicate that thyroid cancer is the seventh most common cancer worldwide, and its incidence is rapidly increasing.^[^ ^6^ ^]^ This rise can be attributed to two primary factors: environmental pollution and prolonged exposure to ionizing radiation, which contribute to a genuine increase in thyroid cancer prevalence, and the enhanced detection rate facilitated by more sensitive diagnostic tools and advanced examination techniques, as shown in Figure 1A.^[^ ^7^ ^]^

The disparity between the low mortality rate of thyroid cancer and its increasing incidence is partly attributed to potential misdiagnosis and overtreatment of thyroid nodules.^[^ ^8^, ^9^ ^]^ Advances in high‐resolution ultrasound have elevated the detection rates of thyroid nodules from 3% to 7% to an astonishing 20%–76%, leading to the overdiagnosis of low‐risk thyroid papillary carcinoma.^[^ ^10^ ^]^ This has resulted in an increased number of patients who could have been safely monitored, undergoing unnecessary interventions such as thyroid surgery, radioactive iodine therapy, or excessive thyroid hormone treatments.^[^ ^11^, ^12^ ^]^ Such overtreatment has led to postoperative complications, including bleeding, diminished thyroid function, and nerve damage, significantly impacting patients' postoperative quality of life.^[^ ^13^ ^]^ The surge in thyroid nodule incidences places considerable pressure on healthcare systems worldwide, particularly in China, making the early and accurate diagnosis of malignant thyroid tumors and their rational treatment a significant global healthcare concern.^[^ ^14^, ^15^ ^]^ Consequently, there is an urgent need for improved diagnostic strategies for thyroid nodules that balance patient care with the escalating demands on healthcare resources.^[^ ^16^, ^17^ ^]^

Ultrasound imaging of the thyroid gland, while useful, cannot conclusively diagnose thyroid cancer; the malignancy must be confirmed through cytological analysis, as shown in Figure 1A. International guidelines recommend that nodules with elevated Thyroid Imaging Reporting & Data System (TI‐RADS) scores should be evaluated using Ultrasound‐Guided Fine‐Needle Aspiration Cytology (FNAC), which is considered the gold standard for thyroid cancer diagnosis due to its high sensitivity and specificity in Figure 1A.^[^ ^18^, ^19^ ^]^ On the pathological front, the Bethesda System for Reporting Thyroid Cytopathology (TBSRTC), in Figure 1B and Figure S1 (Supporting Information), provides a standardized framework for diagnosing thyroid nodules and assessing their Risk of Malignancy (ROM).^[^ ^20^ ^]^ This system categorizes thyroid nodules into six levels, from TBS‐I to TBS‐VI, each associated with a specific ROM, thereby aiding clinicians in making informed management decisions. However, for 15%–30% of nodules classified under TBS III and IV, cytology alone may not definitively classify them as benign or malignant. In such cases, molecular testing (including markers like BRAF, RAS, TERT, PIK3CA) in Figure 1B is often recommended to improve the preoperative assessment of cancer risk and minimize unnecessary surgical procedures.^[^ ^21^ ^]^

The scarcity of skilled and experienced cytopathologists poses a substantial challenge in the field of pathology.^[^ ^22^ ^]^ Cytopathologists, responsible for cellular diagnosis, often rely on traditional microscopic visual diagnosis, which can be subject to variability due to individual expertise, emotional state, and fatigue, resulting in inconsistent review outcomes. The extended duration of training required for pathologists, coupled with the high‐risk nature of the work and relatively low compensation, contributes to a worldwide shortfall of professionals in this specialty, particularly in China. The current patient‐to‐pathologist ratio indicates a need for approximately 100,000 pathologists in China, yet only about20,000 qualified professionals are presently available. With the rise of precision medicine, the demand for pathologists has intensified, highlighting the necessity for innovative solutions to alleviate their workload while improving efficiency, accuracy, and overall quality of work.

In recent years, the integration of artificial intelligence (AI) into the medical domain has shown remarkable promise, with some models achieving or even exceeding the proficiency of medical professionals.^[^ ^23^, ^24^ ^]^ In digital pathology, AI‐driven tasks such as detection, segmentation, and classification have shown exceptional performance, achieving clinical application standards in predicting biomarkers for colorectal cancer,^[^ ^25^ ^]^ identifying breast cancer lymph node metastasis,^[^ ^26^, ^27^ ^]^ and screening for TCT cervical cancer.^[^ ^28^ ^]^ There has been significant progress in applying deep learning techniques to thyroid ultrasound and histopathological images, yielding relatively high accuracy in classifying thyroid nodules.^[^ ^29^ ^]^ Despite these advancements, the adoption of AI in cytopathology solutions in clinical diagnosis remains relatively limited. Challenges include reliance on single‐source samples, limited data availability, and poor model generalization capabilities.^[^ ^30^, ^31^ ^]^ A critical challenge remains that datasets used for model training and testing frequently do not encompass the complete range of real‐world variability, and robust solutions for effectively generalizing optimized models across diverse clinical centers are urgently needed. Current AI research in this area has primarily focused on the benign vs malignant classification of thyroid nodules based on microscopic imaging, without a thorough examination of the specific cytopathological features of thyroid cells, such as the shape of the nuclear membrane, the texture of the nucleus, the nuclear groove, the pseudo‐inclusion, etc.^[^ ^32^, ^33^ ^]^ Moreover, very few studies have successfully incorporated the diagnostic logic outlined by the TBSRTC standard directly into the reasoning mechanisms of AI models, leaving most current systems in the experimental phase and limiting their widespread clinical application.

Researchers and developers usually underappreciate that the generalization capabilities of the model may be influenced by FNAC preparation, FNAC staining methods, and WSI imaging of scanners from different manufacturers, which limit their clinical applicability.^[^ ^34^ ^]^ Image Appearance Migration (IAM)^[^ ^35^ ^]^ is a solution to mitigate the images into a standard feature space and minimise WSI colour variations from different staining protocols, scanners, and acquisition conditions. While IAM methods have been successful in histological tissue analysis in our previous work,^[^ ^36^ ^]^ their direct application to cytology poses unique challenges. These challenges arise from fundamental differences between tissue and cytology samples, including cellular morphology, staining properties, and spatial context. Unlike histological tissue sections, cytological samples consist of dispersed cells without a well‐defined extracellular matrix, leading to staining variability, morphological heterogeneity, and background complexity. Conventional image patch normalization, such as Macenko's^[^ ^37^ ^]^ and Reinhard's^[^ ^38^ ^]^ methods, assumes relatively uniform tissue structures and fails to adapt to the dynamic staining conditions in cytological smears, potentially introducing additional artifacts, variabilities, and misinterpretation in automated analysis. Given these limitations, there is an urgent need for cytology‐specific IAM approaches that incorporate WSI‐level adaptive appearance migration, multi‐scale learning, and self‐supervised techniques to enhance the robustness of computational cytopathology and facilitate accurate diagnostic automation.

To address the above challenges in thyroid cytopathological diagnosis, we introduce a novel AI model named AI‐Thyroid Fine Needle Aspiration (AI‐TFNA), leveraging the strengths of both deep learning and machine learning technologies as illustrated in Figure 1C. This model is meticulously designed to align with The Bethesda System (TBS) standards, aiming to substantially improve the efficiency and accuracy of diagnosing and managing thyroid nodules. To validate the model's effectiveness, a comparative experiment involving junior and senior pathologists has been conducted to illustrate AI‐TFNA's superior efficiency and accuracy. AI‐TFNA is built upon a large‐scale, meticulously annotated dataset derived from thyroid FNAC slides collected from three leading medical institutes in China, as shown in Figure 1C. The model framework comprises three integral components: SEG‐DETECT for lesion segmentation and morphological feature extraction of nuclear, VAN‐tiny for lesion classification and feature extraction, and XGBoost for final classification, all of which are fine‐tuned to classify thyroid lesions in accordance with the TBSRTC criteria. To bolster the model's ability to generalize across various samples, AI‐TFNA was trained on slides prepared and stained through diverse methodologies, and it has the ability to predict BRAF mutations. Comprehensive internal and external datasets, as in Figure 1D,E, were collected and organized to develop, optimize, and generalize an AI‐TFNA model and validate its sensitivity and robustness, indicating its potential for seamless integration into clinical workflows for thyroid nodule diagnosis. This innovative approach holds promise for significantly enhancing diagnostic precision and treatment strategies for thyroid nodules. What's more, we proposed the image appearance migration (IAM) module to quantify and reduce the color variation in the multi‐center validation. IAM is increasingly recognized for its vital role in computational pathology, and ongoing research continues to expand its applications, enhance the robustness of AI pathological diagnostic models, and accelerate its adoption in healthcare systems.

## 2 Results

### 2.1 Comprehensive Experimental Design and Diverse Data Collection for Robust Model Development and Validation

The study's experimental design and the model development pipeline, spanning from sample acquisition to final validation, are depicted in Figure 1C. Between September 2023 and July 2024, we amassed thyroid liquid‐based cytology slides from the pathology departments of three medical centers to serve as the training and validation datasets for developing the AI‐TFNA system. These centers included Southern Medical University's Nan Fang Hospital (SMUH), Zhengzhou University First Affiliated Hospital (ZUFAH), and Peking University Shenzhen Hospital (PUSH). In total, 4,421 thyroid cytology slides were collected to train the AI‐TFNA system, and another 13,939 slides were used for internal validation purposes.

**Figure 2:**
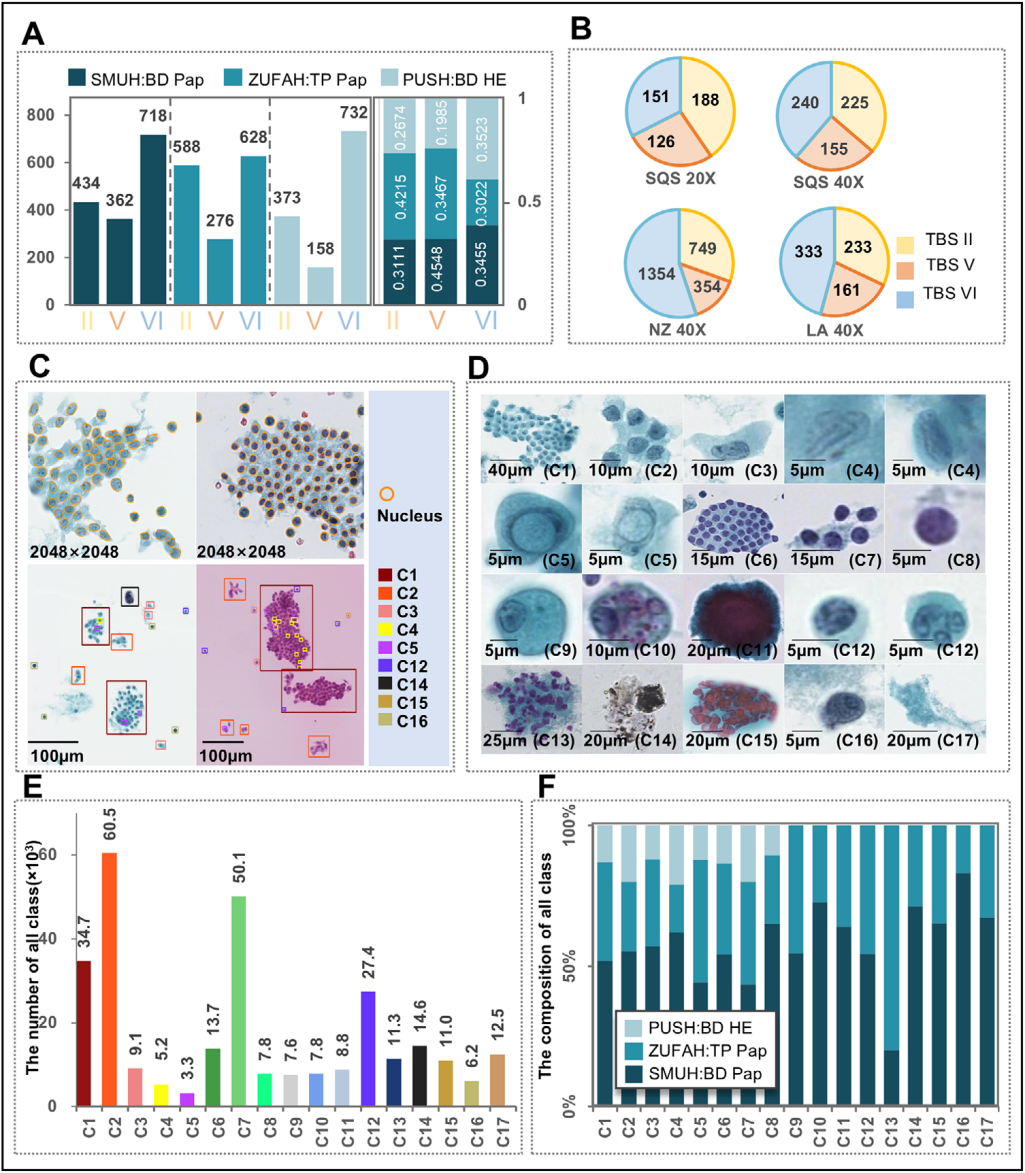
Data preparation and annotation. A). Distribution of thyroid nodules FNAC smears prepared and stained using different protocols collected from three hospitals (*n* = 4269), including BD Pap in SMUH, TP Pap in ZUFAH, and BD HE in PUSH. B). Distribution of collected samples scanned in different scanners and magnification: SQS 20X, SQS 40X, NZ 40X, LA 40X. C). Representative images showing two annotation methods: curve marking of the boundary of each nucleus for training segmentation models (top) and rectangular box selection for training classification models (bottom). D). Representative images of 17 cell types (C1–C17) according to the TBSRTC standard, which served as the classification indices during the learning process. E). The number of cell annotations for the 17 cell types. A total of 291,502 ROIs were obtained for developing the cell classification model. F). The composition of each cell category in images collected from SMUH, ZUFAH, and PUSH. BD Pap: BD SurePath system with Papanicolaou stain; SMUH: Southern Medical University's Nan Fang Hospital; TP Pap: ThinPrep with Pap stain; ZUFAH: The Zhengzhou University First Affiliated Hospital; BD HE: BD SurePath with Hematoxylin and Eosin stain; PUSH: Peking University Shenzhen Hospital; SQS: Shengqiang Scanim; 20X: 20 magnifications; 40X: 40 magnifications; NZ: Hamamatsu NanoZoomer; LA: Leica Aperio.

To account for the variability encountered in real‐world settings and ensure the generalization power of our model, each medical institution implemented distinct slide preparation and staining techniques. This approach allowed for a more robust assessment of diagnostic tools across varying conditions, enhancing their applicability and effectiveness in diverse clinical environments. Particularly, SMUH utilized the BD SurePath system with Papanicolaou (Pap) stain (BD Pap); ZUFAH opted for ThinPrep with Pap stain (TP Pap); and PUSZ used BD SurePath with Hematoxylin and Eosin (H&E) stain (BD HE). Then, we digitalized the samples as shown in Figure S2A (Supporting Information), the details in Section I (Supporting Information). Another layer of variation was introduced in the slide scanning process, where prepared slides were scanned at two different magnifications: 20× and 40×. These variations, along with the TBS (The Bethesda System) classifications from each hospital, are illustrated in **Figure** **2**A,B.

Furthermore, we collected 2,153 clinical samples from four medical institutions from August 2024 to August 2025, in order to conduct an external validation of our system: Fujian Cancer Hospital (FCH), West China Hospital, Sichuan University (WCH), Affiliated Hospital of Nantong University (AHNU), and Huayin Health Medical Group (HHMG). They opted for TP Pap or BD Pap with different scanners (Figure S2C,D, Supporting Information). The detail in Section I (Supporting Information). The distribution of internal and external datasets is detailed in Figure 1D,E.

### 2.2 Annotation Strategy and Extensive Datasets Preparation for Nucleus Segmentation and Cell Classification Models

Out of the total 4,269 WSIs (except TBSI), pathologists performed cell nucleus and cell annotation on 1,440 WSIs, as detailed in Section II(Supporting Information). These annotated slides were used for the development of nucleus segmentation and cell classification models, providing a substantial dataset to train and refine these crucial diagnostic tools. In the cell nucleus annotation, for a given image patch, all cell nucleus masks were annotated as shown in Figure 2C‐top. A total of 2,600 exhausted annotated image patches were obtained for the development of a nuclei segmentation model, including 73,368 nuclei and 6,948 cell clusters. In the cell annotation, cytopathologists identified ROIs in the WSI that contain either a single cell or a cluster of cells of the same category, shown in Figure 2C‐bottom.

Cell annotation included 17 categories as provided in Table S1 (Supporting Information), and the representative images of different classes are illustrated in Figure 2D. These data were obtained in three ways and shown in Figure S2B (Supporting Information): two annotation methods included manual annotation and semi‐annotation via YOLOv5 & SEG‐DETECT, and then we used color normalization to extend the datasets. The corresponding class was then assigned to ROIs. The detailed distribution of annotations and classes is presented in Figure 2E; a total of 291,502 ROIs were obtained for the development of the cell classification model, 256,858 ROIs were used for training, and 34 644 for validation. The above cell annotation data originated from SMUH, ZUFAH, and PUSH; these data details were illustrated in Figure 2F. The remaining 2,829 WSIs with the WSI‐level labels were used for the development of the diagnostic model.

### 2.3 Modular AI Architecture for Robust Cell Detection, Segmentation, and Classification in Thyroid Cytology

**Figure 3:**
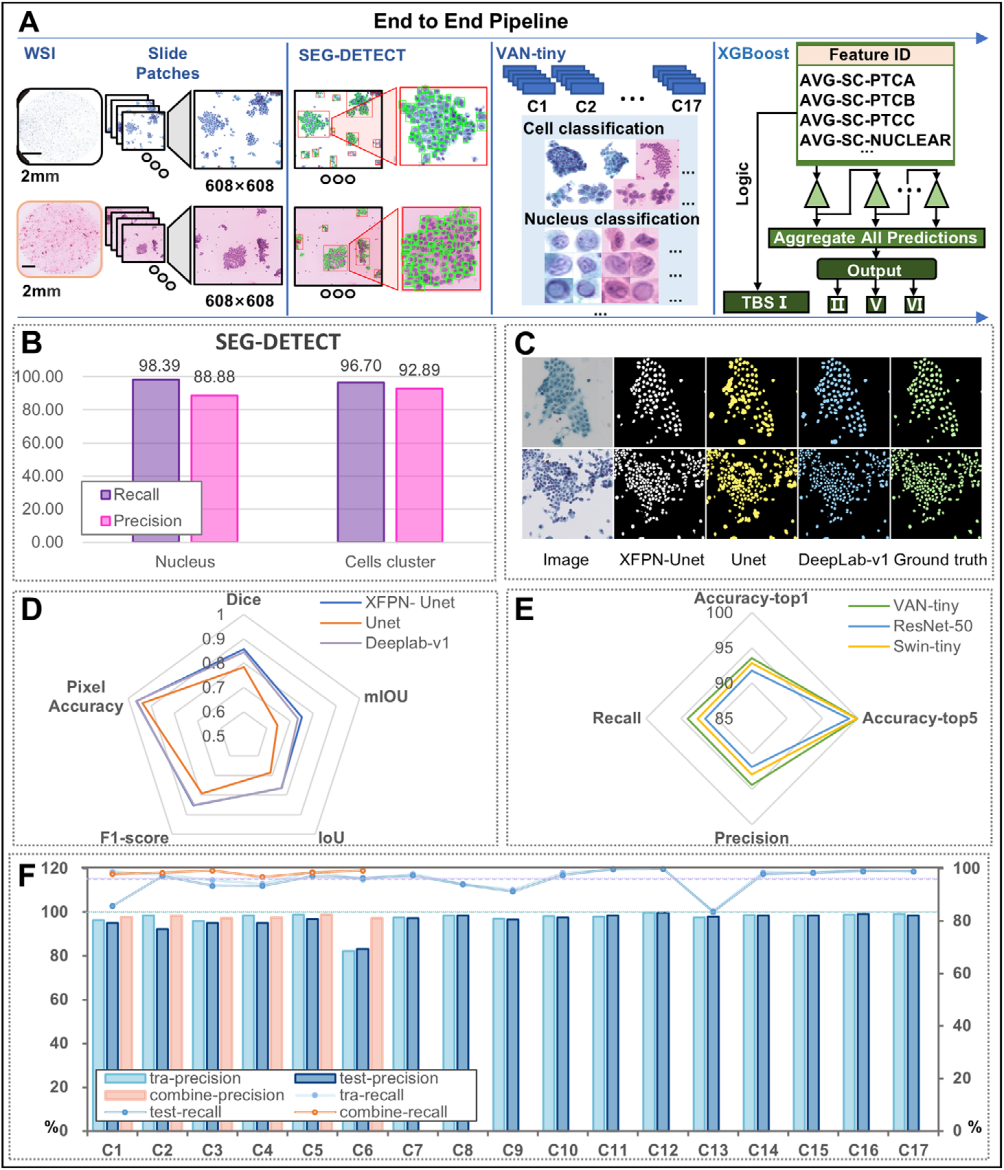
AI‐TFNA model pipeline, cell segmentation and classification model design, and performance. A). The AI‐TFNA model consists of four parts: the SEG‐DETECT model for nuclear segmentation, the VAN‐tiny model for the cell and nucleus classification, the TBS I decision logic, and XGBoost fused statistical features for the slide classification. B). The precision and recall of the SEG‐DETECT model in nuclei and cell clusters. C). Representative image tested using three different nuclear segmentation models (XFPN‐Unet, Unet, and Deeplab‐v1) compared with the ground truth annotations. D). Assessment results of three different segmentation models: XFPN‐Unet, Unet, and Deeplab‐v1 models. E). The assessment results of three different classification models: VAN‐tiny, ResNet‐50, and Swin‐tiny. F). The precision and recall of cell classifications in the VAN‐tiny model and VAN‐tiny combined with the cell XGBoost model (Bar‐plot: precision; Line‐plot: recall).

Generally, the AI‐TFNA system is composed of three AI modules, as in **Figure** **3**A, i.e., the SEG‐DETECT model for the cell detection module, segmentation module, and classification module, the VAN‐tiny model for cell classification, and finally the XGBoost model for whole slide diagnostic, which will be presented in the next Section. Cells in cytological images often exhibit a high degree of overlap and aggregation, posing a challenge for cell segmentation, which is a crucial step in our work. The performance of these three modules was evaluated individually. As designed in Figure S3A (Supporting Information), the SEG‐DETECT module is a combination of the XFPN‐U‐Net‐based cell segmentation model, image morphological processing, and XGBoost classifier for cell classification, enabling precise target localization, high target detection recalls, and preliminary cell classification results. In Figure 3B, the single‐cell recall reached 98.39% and cluster‐cell recall reached 96.70%, which is expected as cluster cells are often more challenging. During SEG‐DETECT module development, three cell segmentation models, using XFPN‐U‐Net, U‐Net, and DeepLab‐v1 architectures, respectively, were trained and evaluated. Compared to the other two models, XFPN‐U‐Net demonstrates better capability in segmenting closely adjacent and aggregated cells. Some of such cases and segmentation results are shown in Figure 3C. In comparison to the U‐Net model, XFPN‐U‐Net outperforms on most metrics while having fewer parameters. As shown in Figure 3D, compared to DeepLab‐v1, XFPN‐U‐Net achieved comparable performance with only 20% of the weight, significantly accelerating the training process and enhancing segmentation efficiency.

Cell classification plays another crucial role in our system design. The model selection and optimization process involved evaluating VAN‐tiny, ResNet50, and Swin‐tiny networks.^[^ ^39^, ^40^ ^]^ The performance comparisons of these three models are presented in Figure 3E. VAN‐tiny was ultimately selected due to its superior performance compared to the other two models, demonstrating its effectiveness in handling the specific needs of cell classification tasks in our system. The details of VAN‐tiny are shown in Figure S3B (Supporting Information). After about227 training epochs, the training loss curve of the VAN‐tiny model gradually stabilized, and the optimal accuracy was achieved, as shown in the zoomed region in Figure S3C (Supporting Information).

Through visualization of testing cell classification results with the confusion matrix, as shown in Figure S4A,B (Supporting Information), we observed that the accuracy of the VAN‐tiny cell classification model for all 17 categories reached 95.57% at the optimal iteration in validation sets. This indicates that the model can accurately differentiate between benign and malignant cells. The model exhibits high precision and recall for each category in Figure 3F, making it crucial for accurately distinguishing various cell types and contributing to the AI‐TFNA system's assessment of the benign or malignant nature of thyroid nodules. Particularly, the model achieved rates of over 92.29% precision and over 85.75% recall for atypical thyroid follicular epithelial cells and over 83.24% precision and over 93.88% recall for normal follicular cells in validation sets in Figure S4B (Supporting Information). VAN‐tiny achieved a high classification recall and precision for nuclear grooves (C4: 93.34%, 95.13%) and pseudo‐inclusions (C5:97.11%, 96.85%). In addition, in order to improve the results of cell classification, we used the cell XGBoost model in the SEG‐DETECT module and the VAN‐tiny model to test C1–C3 and C6–C8 classes again, and only the cells with the same class of the two models entered the next step. The accuracy of the six classes reached 98.28% in Figure S4C (Supporting Information).

Both the SEG‐DETECT model and the VAN‐tiny model demonstrate strong capabilities in cell detection, segmentation, and classification. Specifically, they can effectively differentiate atypical thyroid follicular epithelial cells from normal thyroid cells, which are crucial steps in our research.

### 2.4 WSI‐Level Diagnosis with XGBoost Integrating Cytological Features for TBS Classification of Thyroid Nodules

The diagnostic WSI‐level Classification model based on XGBoost can classify thyroid nodules into II/V/IV categories according to cytologist domain knowledge from TBSRTC diagnostic criteria. In Figure 3A, XGBoost fused morphological and statistical features such as the average area of cells, the number and confidence of different cell classifications obtained by SEG‐DETECT and VAN‐tiny models as its input features (Details given in the Tables S2 and S3, Supporting Information). The grid search strategy was used to find the optimal hyperparameter configuration of the XGBoost classifier, and the WSI‐level XGBoost classifier was trained by 5‐fold cross‐validation.

**Figure 4:**
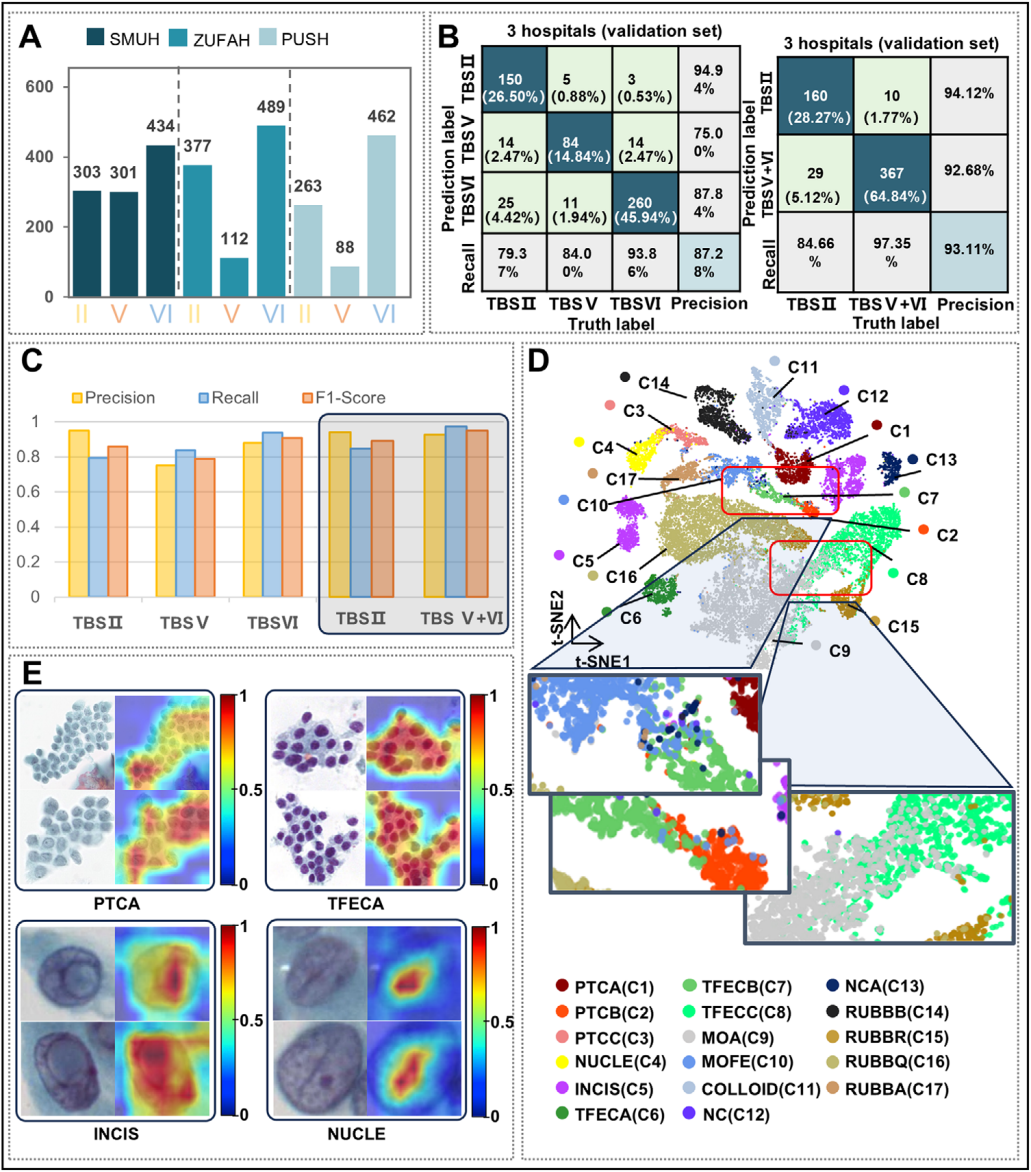
XGBoost model and interpretation of VAN‐tiny. A). Distribution of samples used in the WSI XGBoost model (*n* = 2,829). B). According to the TBSRTC, we trained the WSI XGBoost model to output three diagnostic labels (TBS II/TBS V/TBS VI). In clinical practice, both TBS V and TBS VI indicate malignant thyroid nodules, recommending patients for surgical treatment. Therefore, we trained one another XGBoost model to output two diagnostic labels (TBS II and TBS V+VI). The confusion matrix shows the accuracy of the XGBoost model in the validation set. C). The precision, recall, and F1‐Score in XGBoost for three‐class classification (TBS II, TBS V, and TBS VI) and two‐class classification (TBS II and TBS V+VI) in the validation set. D). The t‐SNE diagram showing the cluster structure and similarity of 17 cell types. E) Heat maps indicating the model's areas of interest to cells in PTCA, TFECA, INCIS, and NUCLE. (Red: more interest; Blue: less interest).

According to the TBSRTC guidelines for malignant risk assessment and clinical management recommendations of thyroid nodules, TBS V and TBS VI often require surgical treatment, while TBS I and TBS II nodules are generally considered benign lesions. Therefore, we trained two independent WSI‐level XGBoost classifiers using 2,829 WSIs, as in **Figure** **4**A: one model for three‐class classification (TBS II, TBS V, and TBS VI) and another model for two‐class classification (TBS II, TBS V+ TBS VI). The training and validation results are shown in Figure 4B and Figures S5 and S6 (Supporting Information). In general, the performance of the two‐class XGBoost classifier is better than that of the three‐class XGBoost classifier. In the two‐class model, the accuracy in distinguishing benign and malignant thyroid nodules reached 93.11%, and the precision and recall of malignant nodules were 92.68% and 97.35%, respectively. In Figure 4C, the results of the three classes showed that the performance of the XGBoost classifier was inferior in TBS V compared with TBS II and TBS VI. The main reason may be that the features extracted from the image are not distinctive enough for the model diagnosis in the training and test process, and the label of TBS V is susceptible to subjective factors of cytopathologists.

### 2.5 Model Interpretability and Visualization Reveal Insights into Cellular Relationships and Diagnostic Cues

t‐SNE is an effective visualization technique that illustrates cell clustering and intercellular relationships within deep learning models. As shown in Figure 4D, the t‐SNE plot revealed a close association between C2 and C7 cells, highlighting the similarities between atypical and normal thyroid follicular epithelial cells. These similarities posed significant challenges in differentiating between these two classes, both in clinical practice and during the development of AI‐TFNA models. Furthermore, recognizing nuclear grooves and pseudo‐inclusions—key cellular features in diagnosing papillary thyroid carcinoma—has been integral to enhancing the model's ability to distinguish atypical cells. The t‐SNE plot also demonstrated the relationships between thyroid follicular epithelial cells and macrophages (C7 with C10, C8 with C9), noting that transformations in thyroid cells, such as eosinophilic or cystic changes, can increase cell size and mimic the appearance of macrophages, in this case, based only on the patch images of the cell without information about the surrounding environment, it's hard for cytopathologists to tell them. These challenges were shown in Figure S7 (Supporting Information). To address these challenges, pathologists have manually annotated a large dataset for training the VAN‐tiny model.

The heatmap visually represented the VAN‐tiny model's focus areas: hotter zones indicated high attention, and colder zones, low attention. In Figure 4E, the heatmap showed that the model primarily targeted nuclear membrane folds in nuclear grooves and the cytoplasm within cell nuclei in pseudo‐inclusions, which were critical for accurate identification. This attention pattern closely mirrored the diagnostic focus of cytopathologists. Additionally, nuclei with a uniform texture appeared to attract more attention than “pale nuclei” among thyroid follicular epithelial cells. The results from the t‐SNE and heatmap analyses align well with the clinical diagnoses made by pathologists, effectively demonstrating the interpretability of the AI model.

### 2.6 Integrating Decision Rules for Quality Control and Enhancing Diagnostic Accuracy through Slide Preparation Assessment

The diagnostic model of AI‐TFNA based on WSI consisted of two steps: the decision rules of TBS I and the XGBoost models for the rest of the TBS grades. The decision rules of TBS I can effectively detect samples that were undiagnosable due to unsatisfactory slide preparation through calculation aimed to reduce the false‐negative rate in thyroid lesion diagnosis. Therefore, we proposed the TBS I decision tree for the identification of TBS I, allowing for effective quality control during the preparation and scanning stages of thyroid cytology slides.

**Figure 5:**
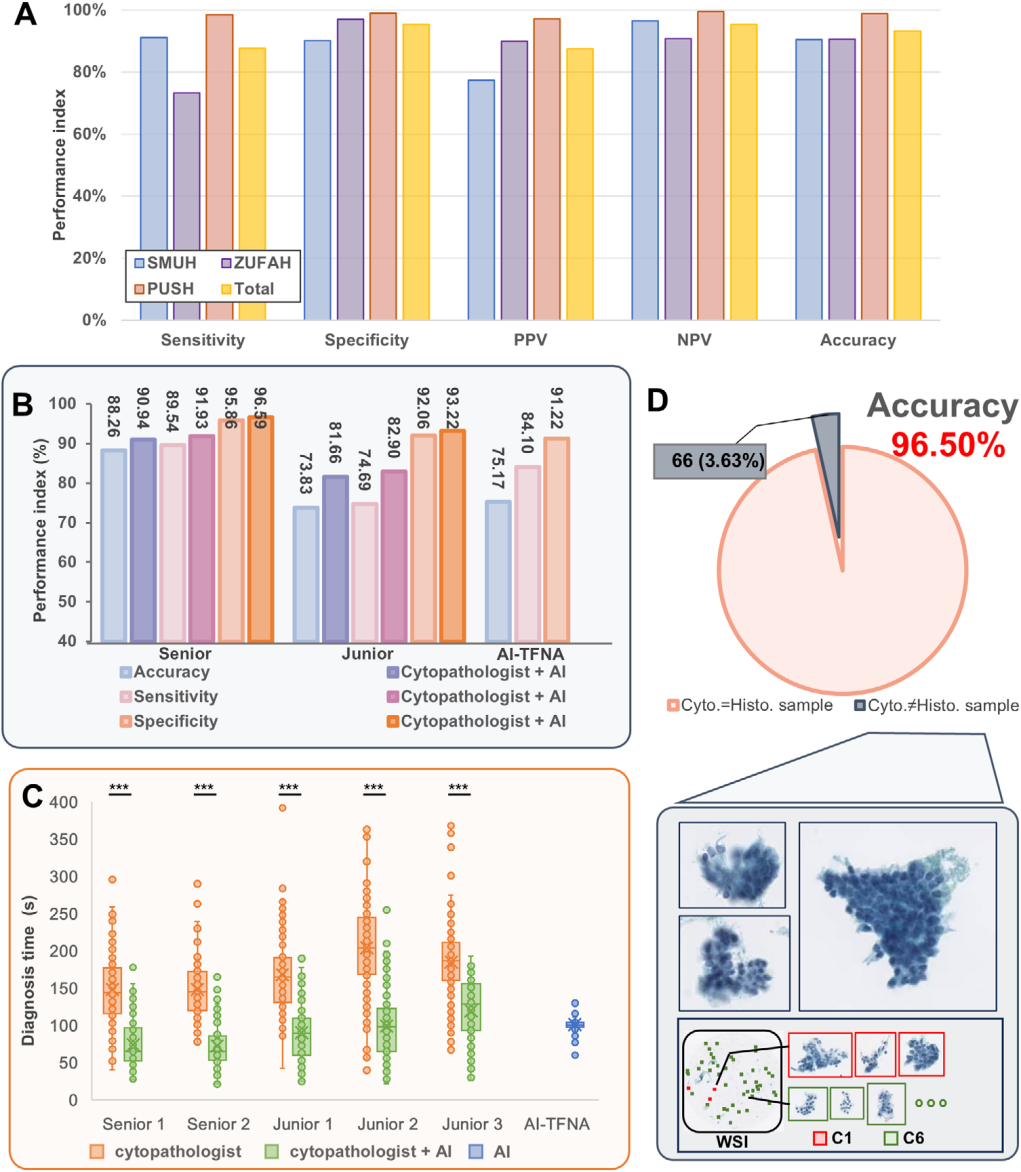
Performance of AI‐assisted diagnosis. A). Accuracy of TBS I diagnosis across three hospitals (SMUH, ZUFAH, and PUSH). Non‐TBS I includes TBS II‐TBS VI. B). Comparison of performance indexes (accuracy, sensitivity, and specificity) among cytopathologists without and with AI assistance, alongside the standalone performance of AI‐TFNA. C). Comparison of diagnosis times among five cytopathologists without and with AI assistance (two‐tailed paired t‐test and Wilcoxon signed‐rank test, *n* = 149, ^***^ *P*<0.001). The boxplot indicates the median (central line), upper and lower quartiles (box limits), and 1.5 × interquartile range (whiskers). D). In Cyto‐Histo Accuracy validation, the cytological results diagnosed by AI‐TFNA with the corresponding histological results showed an accuracy of 96.50%. The primary reasons for AI‐TFNA misdiagnosis were blurred cell scans or an insufficient number of atypical cells in one WSI. SMUH: Southern Medical University's Nan Fang Hospital; ZUFAH: Zhengzhou University First Affiliated Hospital; PUSH: Peking University Shenzhen Hospital.

For the stage of quality control, the overall accuracy of the TBS I decision tree was 93.27% (95%CI 0.9200–0.9453) in three internal hospitals, as shown in **Figure** **5**A and Table S4 (Supporting Information). The sensitivity rate of SMUH and PUSH were 91.11% (95%CI 0.8862–0.9361) and 98.52% (95%CI 0.9746–0.9958) respectively, but ZUFAH was 73.33% (95%CI 0.6946–0.7721). The experiment results revealed that the identification of TBS I was influenced by the slide preparation method. Due to the specificity of ThinPrep production, which resulted in clumps of lymphocytes remaining on the filter membrane and aggregating on the slides. The model misclassified clumps of lymphocytes as benign thyroid follicular epithelial cells, resulting in increased false positives for TBS I.

### 2.7 AI‐TFNA Improves Diagnostic Accuracy and Efficiency in Comparison with Cytopathologists

An independent onsite validation set of 149 WSIs from thyroid FNAC was used to compare AI‐TFNA diagnostic decisions against five cytopathologists. In Figure 5B and Table S5 (Supporting Information), experimental results showed that the evaluation indices of five cytopathologists had increased with the assistance of AI‐TFNA. The accuracy rate of diagnosis by junior cytopathologists was substantially increased from 73.83% (95%CI 0.6975–0.7790) to 81.66% (95%CI 0.7807–0.8524) (7.83%), and the average accuracy rate of senior cytopathologists increased by 2.68%. The diagnostic accuracy rate of AI‐TFNA was 75.17% (95%CI 0.6823–0.8211) in this dataset, which demonstrates the capability to comprehensively identify pathological cells in WSI while providing visual cell classification, thereby reducing cytopathologists' diagnostic missed diagnosis rate and offering valuable decision‐support reference.

As shown in Figure 5C and Table S6 (Supporting Information), the average diagnostic time of AI‐TFNA was 101.50s/slide, which was shorter than that of senior cytopathologists (149.69 s/slide) and junior cytopathologists (185.79 s/slide). The diagnostic efficiency of five pathologists was significantly improved with the assistance of AI‐TFNA (P<0.001). In Figure 5C, the most outstanding results were obtained by senior cytopathologists. The average diagnostic time was shortened from 149.69 to 73.43 s/slide, and the efficiency was increased by 2.04 times. Meanwhile, the efficiency of junior cytopathologists increased about 1.93 times (from 185.79 to 96.21 s/slide). AI‐TFNA can help cytopathologists improve their diagnostic ability and diagnostic efficiency. It effectively reduced the potential misgrading of thyroid nodules due to subjective factors and shortened the diagnostic period.

### 2.8 Comprehensive Internal and External Validation Confirms the Diagnostic Accuracy and Robustness of AI‐TFNA Across Diverse Clinical Settings

We obtained 1884 TBS II, TBS V, and TBS VI thyroid FNAC slides and the corresponding histological diagnosis results. Comparing the cytological results diagnosed by AI‐TFNA with the corresponding histological results, the accuracy of AI‐TFNA for thyroid nodule lesions was 96.50% (95%CI 0.9567‐0.9733), further confirming the robust diagnostic capability of AI‐TFNA (Figure 5D; Table S7, Supporting Information). In this study, we found that the main reasons for AI‐TFNA misdiagnosis were two reasons: I). Some cell images were blurred, resulting in incorrect classification; II). The number of atypical thyroid follicular cells in WSI was much less than that of normal follicular cells, resulting in the missed diagnosis.

**Figure 6:**
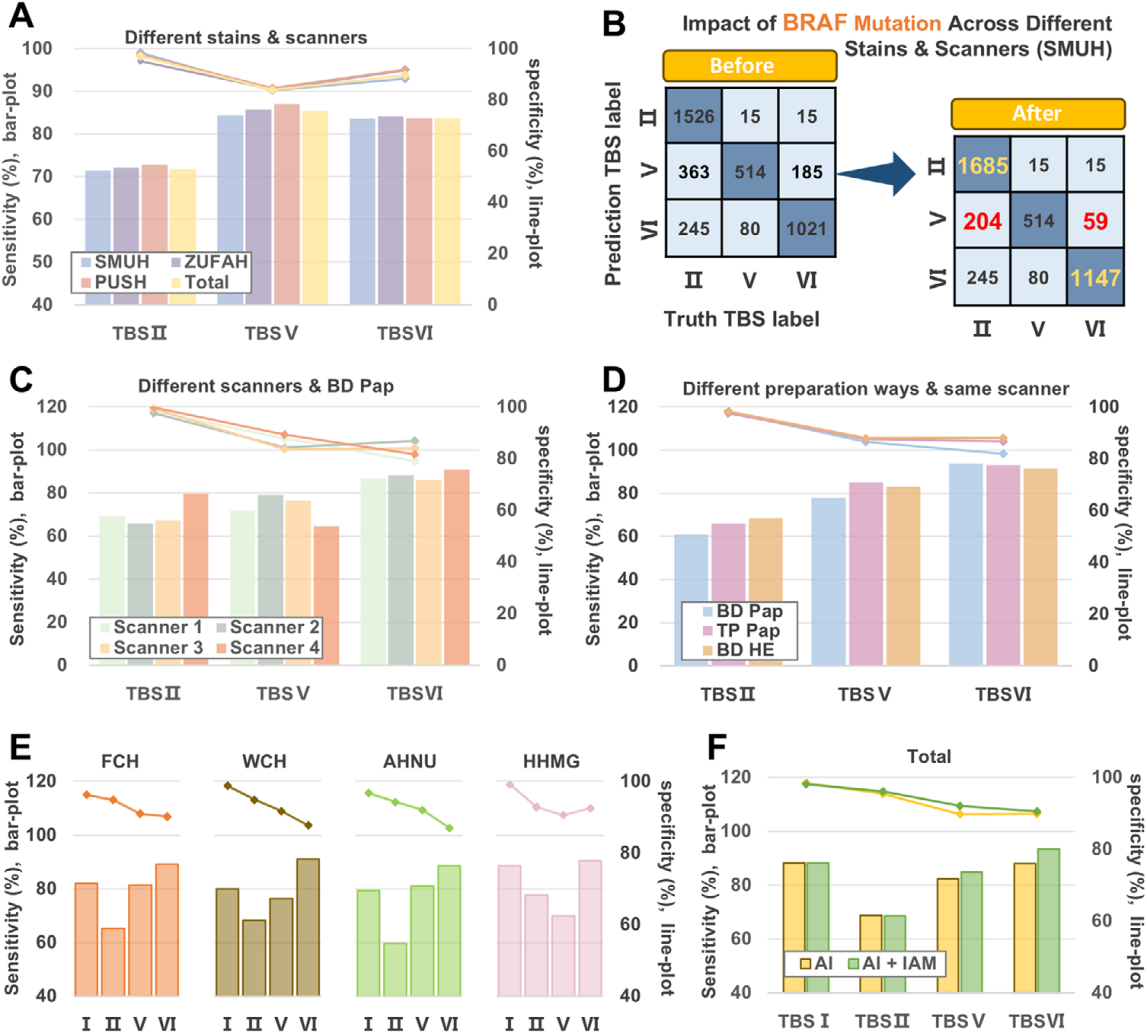
Diagnosis performance of AI‐TFNA and clinical trial. A) Sensitivity and specificity were used to evaluate the performance of AI‐TFNA in the internal datasets (SMUH, ZUFAH, PUSH) with different slide preparation ways, staining schemes, and scanners. B). In the SMUH dataset, 363 TBS II and 185 TBS VI were incorrectly classified as TBS V. 159 TBS II and 126 TBS VI were correctly classified due to the WSI XGBoost model, which combined the BRAF‐V600E gene prediction model. C). The FNAC slides of BD SurePath pap stain (BD Pap) were collected to compare the sensitivity and specificity of four scanners. D). These slides were digitised by NZ 40× and collected to compare the performance of different preparation methods. E). Sensitivity and specificity were used to evaluate the performance of AI‐TFNA in the external datasets (FCH, WCH, AHNU, and HHMG). F). Image Appearance Migration (IAM) to improve the generalizability of AI‐TFNA, the results are in five institutes (ZUFAH, PUSH, FCH, WCH, AHNU). SUMH: Southern Medical University's Nan Fang Hospital; ZUFAH: Zhengzhou University First Affiliated Hospital; PUSH: Peking University Shenzhen Hospital; FCH: Fujian Cancer Hospital; WCH: West China Hospital, Sichuan University; AHNU: Affiliated Hospital of Nantong University; HHMG: Huayin Health Medical Group. BD Pap: BD SurePath system with Papanicolaou stain; TP Pap: ThinPrep with Pap stain; BD HE: BD SurePath with Hematoxylin and Eosin stain.

We totally collected 7,351 WSIs from SMUH, ZUFAH, and PUSH for further validation, including thyroid nodules in TBS II, TBS V, and TBS VI according to TBSRTC. The sensitivities of AI‐TFNA in TBS V and TBS VI were 85.37% (95%CI 0.8357‐0.8717) and 83.78% (95%CI 0.8240‐0.8517) in **Figure** **6**A. In addition, the specificity of TBS II reached 97.13% (95%CI 0.9663‐0.9763). AI‐TFNA showed a desirable performance in a large dataset; the model based only on image information has reached the optimal performance. However, in this dataset, 363 TBS II and 185 TBS VI were incorrectly classified as TBS V in SMUH. We thus trained the Gene XGBoost classifier, which can predict BRAF gene mutation information, and the results are shown in Figure S8 (Supporting Information). WSI‐level XGBoost classifiers were combined with the BRAF mutation prediction model to analyze this data, as shown in Figure 6B, 159 TBS II and 126 TBS VI were correctly classified. Therefore, it is very meaningful to construct a multi‐modal fusion AI model for the judgment of benign and malignant thyroid nodules. In Figure 6C,D and Table S8 (Supporting Information), we analyzed the performance of the AI‐TFNA system, and the data indicated that AI‐TFNA had good sensitivity and specificity in different slide preparation methods, staining schemes, and scanners. Among the four scanners evaluated, AI‐TFNA demonstrated superior performance at Scanner 3 (20X) compared to Scanner 1 and 2 (40X). Although performance was marginally better at Scanner 4 (40X, the same mode as Scanner 3). AI‐TFNA remains a powerful tool for thyroid nodule diagnosis in clinical settings where 20X scanning is the standard or preferred resolution.

Our study tested AI‐TFNA's capability using 427 samples of TBS III and 292 samples of TBS IV from four institutions. AI‐TFNA achieved sensitivities of 77.99% for TBS III lesions and 90.75% for TBS IV lesions, highlighting its potential for indicating malignancy in ambiguous cases, as shown in **Table** **1**. In addition, 120 thyroid FNAC samples classified as TBS III and TBS IV that subsequently underwent surgical treatment were included. Histological confirmation revealed that 34 cases confirmed as benign conditions (e.g., nodular goiter or thyroiditis) indicate incorrect diagnosis and treatment. AI‐TFNA correctly reclassified 4 cases of these histologically benign cases as TBS II, demonstrating its potential to reduce unnecessary medical procedures by mitigating overdiagnosis and overtreatment.

**Table 1:** Diagnosis of AI‐TFNA in TBS III and TBS IV.

Samples	Number	Medical Institution	The predicted result of AI‐TFNA				Positive sample number	Missed sample number	Sensitivity [95% CI]
			TBSI	TBSII	TBSV	TBSVI			
TBS III	427	SMUH	8	27	122	27	333	94	77.99% (0.7406‐0.8192)
		ZUFAH	0	0	2	1			
		PUSH	8	17	82	28			
		HHMG	0	34	56	15			
TBS IV	292	SMUH	0	12	31	57	265	27	90.75% (0.8743‐0.9408)
		ZUFAH	0	1	0	1			
		PUSH	0	13	54	91			
		HHMG	0	1	25	6			
Total	719		16	104	347	220	598	121	83.17% (0.8044‐0.8591)

SMUH: Southern Medical University's Nan Fang Hospital; ZUFAH: Zhengzhou University First Affiliated Hospital; PUSH: Peking University Shenzhen Hospital. HHMG: Huayin Health Medical Group.

In the external samples, we obtained 2016 thyroid FNAC slides from four hospitals in China with different slide preparation methods and scanners. In Figure 6E and Table S9 (Supporting Information), AI‐TFNA showed the sensitivities were 79.54% (95%CI 0.7377‐0.8531), 78.97% (95%CI 0.7301‐0.8492), 77.12% (95%CI 0.7144‐0.8280), and 81.64% (95%CI 0.7964‐0.8364) in FCH, WCH, AHNU, and HHMG, respectively. The specificities were 93.10% (95%CI 0.8947‐0.9672), 93.33% (95%CI 0.8968‐0.9697), 92.53% (95%CI 0.8897‐0.9608), and 93.80% (95%CI 0.9256‐0.9505), respectively.

### 2.9 Further Enhancing Model Generalization with Image Appearance Migration for Robust Cross‐Institutional Diagnostic Performance

Model generalization is a critical consideration—and a major bottleneck—for the clinical adoption of AI diagnostic systems. Generalization hinges on two key factors: i) variability in image appearance caused by differences in sample preparation and scanning processes, and ii) variability in cellular patterns across different cell types. The concept of enhancing model generalizability through image appearance adaptation is based on the premise that such appearance variations are both quantifiable and compensable. By addressing these differences, model performance can be stabilized across diverse datasets, thereby improving its robustness in real‐world clinical settings.

To evaluate the generalizability of the AI‐TFNA model across institutions with different sample preparation protocols—including variations in staining methods, cell sedimentation techniques, and whole‐slide imaging scanners—we employed the Image Appearance Migration (IAM) approach for both internal and external validation. The default SMUH dataset, prepared using the BD SurePath system and Papanicolaou (Pap) staining (BD Pap), served as the reference domain for image appearance alignment. This strategy was designed to simulate real‐world deployment scenarios and assess the robustness of the AI‐TFNA model when applied to diverse cytology samples. The pseudocode for the IAM process is provided in Section III (Supporting Information), and further implementation details can be found in Figures S9 and S10 and Table S10 (Supporting Information). As shown in Figure 6F and Figure S11 (Supporting Information), the IAM‐enhanced model demonstrated consistent improvements in both sensitivity and specificity across multiple datasets, indicating its potential to achieve stable performance and generalizability despite institutional variability.

The AI‐TFNA model is designed according to TBSRTC, and it is adaptive to sample preparation using different staining protocols and scanners with strong generalization. It is expected to be applied in clinical work and play an important role in daily diagnostic procedures for thyroid nodules.

## 3 Conclusion and Discussion

Traditionally, thyroid nodule diagnosis relies heavily on manual microscopic examination by pathologists—a labor‐intensive, subjective process vulnerable to diagnostic variability due to differences in pathologists' expertise and limited quality control.^[^ ^41^ ^]^ The complexity of thyroid cytology slides underscores the urgent need for a standardized, precise, AI‐assisted diagnostic approach,^[^ ^42^ ^]^ which AI‐TFNA successfully addresses. The AI‐TFNA system demonstrates remarkable efficacy in discriminating between benign and malignant thyroid nodules and classifies the samples according to the TBS standard, providing valuable diagnostic support to cytopathologists. Our results confirm that the AI‐TFNA system achieves high accuracy and strong generalization capabilities, positioning it as a promising tool for widespread clinical adoption. By integrating deep learning solutions, AI‐TFNA significantly enhances the efficiency and precision of thyroid nodule diagnosis, ultimately contributing to improved patient outcomes.

A key strength of our study includes the extensive dataset collected from six hospitals, which ensures diversity and comprehensiveness by incorporating real‐world variations. This large‐scale data collection accounts for differences in slide preparation, staining protocols, and scanning techniques, which are crucial for developing a robust AI model effective across varied clinical settings. In our development process, we encountered challenges such as distinguishing benign “papillary hyperplasia” from papillary structures indicative of thyroid carcinoma. Technical issues with the ThinPrep Pap protocol and minimal cytoplasm‐to‐nuclei color contrasts also presented difficulties. These were mitigated through strict quality control measures in slide preparation, staining, and scanning procedures, combined with increasing the volume and diversity of training data, thus significantly enhancing AI‐TFNA's diagnostic performance. Such meticulous data handling facilitates the optimization and generalization of the AI‐TFNA system, enabling it to maintain high performance not only on internal datasets but also on external validations. Advanced AI techniques leveraged by our model, such as deep learning‐driven feature extraction at multiple levels, were essential in achieving exceptional diagnostic capabilities.^[^ ^43^ ^]^ Our findings align with previous studies, further validating the reliability and consistency of our approach.^[^ ^28^ ^]^

In computational pathology, the foundation models have recently been developed to improve the general representation of pathology‐specific features for classification and diagnosis. Notable models such as CLAM,^[^ ^44^ ^]^ UNI,^[^ ^45^ ^]^ CONCH,^[^ ^46^ ^]^ and CHIEF^[^ ^47^ ^]^ have demonstrated significant success in histopathology by leveraging weakly supervised learning, self‐supervised learning, and standardized benchmarking to enhance model performance and generalizability. However, foundation models for cytology remain underdeveloped due to unique challenges posed by cellular morphology, overlapping structures, and limited datasets. Our AI‐TFNA system addresses these challenges by introducing robust AI methodologies adapted specifically to cytology, enhancing diagnostic accuracy and clinical applicability. Future efforts should further adapt these foundation model methodologies to cytological analysis.

Clinically, AI‐TFNA significantly addresses China's challenge of imbalanced healthcare resources and the critical shortage of qualified pathologists. Designed in strict accordance with the TBSRTC diagnostic standards from the cell annotation stage to final decision‐making, the AI‐TFNA model integrates cell quantity, nuclear membrane morphology, texture, and other relevant characteristics to improve accuracy and clinical applicability. Particularly, the VAN‐tiny model component of our system effectively resolves complex clinical scenarios, distinguishing benign from malignant thyroid follicular epithelial cells and differentiating between follicular epithelial cells and macrophages with precision and recall rates exceeding 90%. Despite AI‐TFNA's superior capabilities in some diagnostic respects, its primary role remains supportive, assisting pathologists rather than replacing human expertise. The system notably distinguishes TBS V lesions as “suspicious for malignancy” from “malignant” TBS VI, thereby guiding clinicians toward appropriate surgical decisions.

A novel aspect of our study is the introduction of Image Appearance Migration (IAM) into cytological image analysis for the first time. IAM is an innovative technique that standardizes and harmonizes pathology images from heterogeneous sources by addressing significant challenges posed by diverse scanners, staining protocols, and lab conditions. Its application in cytology is particularly noteworthy, given the complexity of cellular morphology and overlapping cellular structures inherent to cytological samples. Our results demonstrate that IAM significantly improves the robustness and generalization of AI‐TFNA, enhancing diagnostic accuracy across different clinical settings. This advancement facilitates more reliable predictions, promotes effective cross‐domain applications, and supports large‐scale data analyses in computational cytopathology. The AI‐TFNA system presents numerous practical advantages, including cost‐effectiveness, shorter processing times, and exceptional diagnostic accuracy across various slide preparations and staining methods. These characteristics make it particularly beneficial in resource‐limited regions where the shortage of trained pathologists poses a severe barrier to quality healthcare. Although the model's performance has improved in Papanicolaou‐stained samples, when applied to Hematoxylin and Eosin (HE) stained samples, the IAM method used here needs to make an adaptation to the H&E staining scenario. It needs to recalculate the tissue mask. The histology and cytology have different structures, so we need to use different tissue mask extraction methods. Otherwise, it may cause image staining distortion due to the inaccurate tissue region extraction.

Despite these strengths, the AI‐TFNA system does have limitations. For instance, due to limited characteristic diagnostic features in cells classified as “suspicious for malignancy” (TBS V), the XGBoost diagnostic model demonstrated lower accuracy in these cases. Furthermore, subjective annotations by pathologists influenced model performance, underscoring the need to expand training datasets further. This aligns with clinical realities in some institutions, where the ambiguity between categories TBS V and VI could lead to conservative classifications intended to minimize potential medical disputes. Looking ahead, integrating additional modalities such as patient clinical history, imaging data, histopathology images, and molecular profiles could further improve the diagnostic accuracy of AI‐TFNA. In addition, highlighting which cellular features (e.g., nuclear contours, chromatin patterns) most influenced the AI's prediction and providing its confidence scoring to flag ambiguous cases for cytopathologist review can improve the diagnostic accuracy of TBS V.

## 4 Experimental Section

### AI‐TFNA System Development

AI‐TFNA was designed strictly according to TBSRTCI‐VI to assist pathologists in the diagnosis of thyroid nodules. This system was composed of three AI models: the cell detection and segmentation SEG‐DETECT model, the cell classification VAN‐tiny model, and the diagnostic model. The diagnostic model consisted of two steps: the decision rules of TBS I and the AI models for the rest of the TBS grades. First, the specific criteria for the TBS I class based on the features extracted from SEG‐DETECT and VAN‐tiny was devised. The specific criteria are mainly based on the number of thyroid follicular epithelium cell mass and the total cell count. Thereafter, a specific formula in online methods was used to calculate whether the WSI met the criteria of TBS I. Then, the XGBoost model was applied for the classification of TBS II, TBS V, and TBS VI (Figure 3A).

Based on the large annotated dataset, we developed the AI‐TFNA system, which consists of the following four parts:

### AI‐TFNA System Development—SEG‐DETECT Detection

In Figure S3A (Supporting Information), the SEG‐DETECT model consists of four core modules: the nuclear segmentation module (XFPN‐U‐Net), the color space transformation module, the image post‐processing module, and the XGBoost cell classification model with morphological feature extraction. First, the nuclear segmentation module was employed to separate nucleus targets from the background. Second, the color space transformation module was used to eliminate some nucleus‐similar debris (such as red blood cells) with the HSV (Hue, Saturation, Value) range, thereby reducing instances of false positives. To achieve more precise localization and a higher object detection recall rate, an image post‐processing module was meticulously designed, in which a lot of bounding boxes of cell clusters and nuclei would be generated and served as the input for the subsequent VAN‐tiny cell classification model inference. Finally, a series of image processing methods was used to extract a total of 19 morphological features of nuclei and train an XGBoost model for cell classification.

### AI‐TFNA System Development—VAN‐Tiny Cell Classification

The self‐attention mechanism, as shown in Figure S3B (Supporting Information), which was initially designed for natural language processing (NLP)^[^ ^48^ ^]^ tasks, had recently shown promising results in the field of computer vision. VAN‐tiny^[^ ^49^ ^]^ leverages the advantages of self‐attention and large kernel convolutions, surpassing CNN and Transformer models significantly in image classification. The model was trained with AdamW optimizer (weight decay = 0.05) and cosine annealing strategy (initial learning rate = 0.001) for 227 epochs to gain the highest performance and generalization, while the input was transformed with a series of argumentation, and randomly cropped to 224 × 224 (Figure S3C, Supporting Information).

### AI‐TFNA System Development—Feature Extraction

The quantities of object detected in each WSI and classified by the VAN‐tiny model as PTCA, PTCB, PTCC, TFECA, TFECB, TFECC, NUCLEAR, INCIS, and other were statistically analyzed to derive features denoted as N‐^*^, along with corresponding average classification probabilities denoted as AVG‐P‐^*^, median classification probabilities denoted as MEDI‐P‐^*^, average nuclear morphological feature donated as AVG‐^*^‐NUCLEAR‐^*^, median nuclear morphological feature donated as MEDI‐^*^‐NUCLEAR^*^, average detection probabilities denoted as AVG‐SC‐^*^, and median detection probabilities denoted as MEDI‐SC‐^*^. These features encompassed a total of 104 statistical attributes, as illustrated in Tables S2 and S3 (Supporting Information). Additionally, clinical diagnostic labels were assigned to each WSI by cytopathologists. Ultimately, the compiled statistical features, along with their corresponding diagnostic labels, constituted a comprehensive feature set utilized for training the XGBoost model.

### AI‐TFNA System Development—WSI Classifier Construction

XGBoost^[^ ^50^ ^]^ ML structure, a powerful gradient boosting tree framework, to train a diagnosis model, which can mimic the diagnostic reporting process of pathologists, with features extracted was used. For the training, the Hyperopt library was utilized to perform a grid search in the hyperparameter space, based on the results of 5‐fold cross‐validation.

As can be seen in Figure 3A, the SEG‐DETECT model detected ROIs in WSI, and the VAN‐tiny model classified the ROIs output by the SEG‐DETECT model. The WSI diagnostic model consisted of two components: the decision rules of TBS I and the XGBoost diagnostic model. The formula according to 152 TBS I WSIs was designed, when WSIs meeting the specified criteria based on features extracted from SEG‐DETECT and VAN‐tiny will be categorized into the TBS I class:

(1) $$\begin{array}{ccc} \mathrm{AVG}\_A & & *\left(N-\mathrm{PTCA}\;+\;N-\mathrm{TFECA}\right)+\mathrm{AVG}\_B\\ & & *\left(N-\mathrm{PTCB}\;+N-\mathrm{TFECB}\right)<\mathrm{GLOB}\_\mathrm{CELL} \end{array}$$

where *AVG_A* denotes the average cell count of PTCA and TFECA, while *AVG_B* represents the average cell count of PTCB and TFECB. *GLOB_CELL* indicates the total cell count used to determine if the WSI belongs to TBS I, empirically set at 60, with *AVG_A* and *AVG_B* set at 10 and 5, respectively. If the WSI is not classified as TBS I, the XGBoost model is employed for prediction.

### Model Visualization

Some approaches were used to visualize the VAN‐tiny model. To observe the distance distribution of intra‐class and inter‐class in 17 classes, the output high‐dimensional feature maps were transformed from the VAN‐tiny cell classification model into two dimensions by using the t‐SNE (t‐stochastic neighborhood embedding) method. Besides, GradCAM precisely identifies and highlights areas of the input image that contribute significantly to a particular category of predictions, which helps to have a deeper understanding of the VAN‐tiny model's decisions. In the GradCAM picture, the red area indicated a higher level of attention from the model, reflecting its greater contribution to the final prediction result. Conversely, the model pays less attention to the yellow part of the image, while the blue features have minimal influence on the model.

### The Validation of the AI‐TFNA Model

After the model was built, a comprehensive internal validation including 13939 thyroid FNAC WSI was performed to evaluate the performance of the model in diagnosability and generalizability. There were five categories of the internal validation in Figure 1D: 1) Pre‐requirement detection validation: The testing set included 405 TBSI and 1095 TBSII‐VI, which were used to test whether AI‐TFNA could distinguish samples that could be diagnosed from those that could not. 2) AI‐Human Accuracy validation: An independent testing set of thyroid FNAC was collected from SUMH, 149 WSIs were diagnosed by the AI‐TFNA model and 5 cytopathologists, respectively. Five pathologists with different levels of clinical experience, 3 junior cytopathologists, and 2 senior cytopathologists were instructed to independently diagnose each WSI of thyroid FNAC according to TBSRTC. Different from the usual clinical work, no clinical information (age, gender, medical history, imageological examination, etc.) of the patient was provided, and the pathologists were given the diagnosis results based on WSI and recorded the diagnosis duration of each WSI. Three weeks later, the five pathologists diagnosed the testing set with the assistance of the AI‐TFNA system and recorded the diagnosis time. 3) Cyto‐Histo Accuracy validation: In addition, 1,884 patients with thyroid FNAC TBSII, TBSV, and TBSVI from SMUH, ZUFAH, PUSH, and their surgical specimens were confirmed as thyroid malignancies by histopathology and were used to test for the diagnostic accuracy rate. 4) The generalization validation of AI‐TFNA: a) 7,351 WSIs from three internal hospitals with different slide preparations, staining techniques, and different scans. b) 1500 WSI included three different slide preparations and stains with the same scanner. c) 2,000 WSI with BD Pap through the digitalization of four different scanners (LA40/NZ40/SQS20/SQS40). 5) Positive detection validation: 427 TBS III and 292 TBS IV were contained in this dataset to evaluate the ability of AI‐TFNA to reduce missed diagnosis.

Finally, in order to further validate the AI‐TFNA model's capacity to diagnose thyroid nodules, from August 2024 to August 2025, samples were collected from FCH, WCH, AHNU, and HHMG for the external validation.

### Construction of Multimodal Fusion Model

290 thyroid nodules (with BRAF‐V600E mutation) were collected. A cascade prediction strategy was used to combine WSI‐XGBoost and Gene‐XGBoost models to give the final diagnosis result of FNAC. When WSI‐XGBoost outputted “TBSV”, Gene‐XGBoost would output “0” or “1”; the final diagnosis results outputted TBSII(TBSV+0) or TBSVI (TBSV+1). Then 548 WSI were tested from Different stains & scanners (SMUH) cohort, which were labeled II or VI, misclassified to V by AI‐TFNA.

### Hardware and Software

The DL models were developed and trained using Python 3.7 on PyCharm (https://www.jetbrains.com/pycharm/). Specifically, the deep learning framework uses Pytorch 1.13.1, and the machine learning framework XGBoost model for diagnostic uses Scikit‐learn 0.21.1 under the Ubuntu 16.04×64 operating system. We used OpenSlide (1.1.2), a C library that provided a simple interface, to read whole‐slide images. All statistical analyses were performed in Python 3.7 and its statistical library consisting of Pandas 1.3.5, Matplotlib 3.5.2, NumPy 1.21.5, and SciPy 1.10.1. The computer specifications used to develop and train AI algorithms are RAM: 32GB, CPU: 12 Intel(R) Core (TM) i7‐8700K CPU @ 3.70 GHz, and GPU: 2 GeForce RTX 2080 SUPER.

### Ethics Approval

The experimental design and methods were performed in accordance with the relevant guidelines and regulations reviewed by the Ethics Committee of Southern medical University Nanfang Hospital (Ref. No. NFEC‐2023‐401), Zhengzhou University First Affiliated Hospital (Ref. No. 2024‐KY‐0864‐001), Peking University Shenzhen Hospital (Ref.[2024] No.102), Fujian Cancer Hospital (Ref. No. K2024‐271‐01), West China Hospital, Sichuan University (Ref.[2024] No.1263), Affiliated Hospital of Nantong University (Ref. No. 2024‐K121‐01) and Guangzhou Huayin Health Medical Group (Ref. LLPJ2025003).

### Consent for Publication

All authors of this work agreed to publish with Advanced Science once accepted.

### Availability of the Data and Materials

In this study, a novel AI diagnostic system called AI‐TFNA was introduced to assist in the diagnosis of TBS classification of thyroid FNAC slides. This paper was produced using no publicly available image data, as it is constrained by personal information protection, patient privacy regulation, and medical institutional data regulatory policies, etc. The size of our research data is also too huge to be properly stored in public repositories. However, the authors have made every effort to make these resources publicly available, such as the source code, software methods, and supporting information to reproduce the technical pipeline, analyses, and results. All data supporting the findings of this work are available unconditionally for accredited scientific researchers for the purpose of reproducing the results and/or further academic and AI‐related research activities from the primary corresponding author dyqgz@126.com upon request within 10 working days.

## Conflict of Interest

The authors declare no conflict of interest.

## Author Contributions

Y.L., Y.S., H.L., W.L., and W.Y. contributed equally to this work. X.B., Y.D., and W.Y. contributed to the conceptualization of the study. W.Y., K.O., and Y.L. supervised the research and contributed to project coordination and administration. Y.S., Y.L., and Y.D. contributed to the development or design of the methodology. W.L., W.Y., G.C., Y.J., X.Y., Y.L., Z.Z., S.L., and X.W. contributed to the data annotation and investigation. X.H., M.Y., N.Z., and L.L. contributed to resources and supervision. H.Z. and B.S. contributed to the experiment design, the development of the abnormal cell detection model, and the integration of the AI system. Y.S. and H.Z. contributed to the development of the SEG‐DETECT model. X.Z. and H.Z. contributed to the development of the cell classification model and WSI‐level classifier. H.L. contributed to the development of the image appearance migration (IAM) module. C.W., W.P., and Y.Z. prepared the manuscript with the contribution of all other authors. H.L., H.Z., and Y.L. contributed to the preparation of Supporting Information. Y.D., W.Y., Y.L., K.O., and H.L. contributed to the review and revision of the manuscript. S.L., L.L., and M.Y. wrote the paper with the assistance and feedback of all the other co‐authors.

## Supporting information



Supporting Information

## Data Availability

The source code for training the models mentioned in this work is available at https://github.com/SUYONGJIAN/AI‐TFNA or obtained by sending a request to the primary corresponding author (Prof. Y.Q. Ding, dyqgz@126.com).
