# Supplementary material for: Cytological Classification Diagnosis for Thyroid Nodules via Multimodal Model Deep Learning
Source: Adv Sci (Weinh). 2025 Oct 16;12(48):e11369. doi: 10.1002/advs.202511369 (PMC12752556; doi:10.1002/advs.202511369)
Supplement: Supplementary file 1 — Supporting Information [file ADVS-12-e11369-s001.docx]

**Cytological classification Diagnosis for thyroid nodules via multimodal model deep learning**

**Supporting Information**

**TABLE OF CONTENTS**

**Cytological classification Diagnosis for thyroid nodules via multimodal model deep learning**

**[1](#_Toc190954550)**

**[Section I: FNAC samples digitization and reviewing 2](#_Toc190954551)**

**[Section II: Datasets annotation and reviewing 2](#_Toc190954551)**

**[Section III: Image Appearance Migration (IAM) 3](#_Toc190954552)**

**[Section Ⅳ: Evaluation metrics](#_Toc190954552) 3**

**Figure S15**

**[Figure S2](#_Toc190954552) 6**

**[Figure S3](#_Toc190954552) 7**

**[Figure S4](#_Toc190954552) 8**

**[Figure S5](#_Toc190954552) 9**

**[Figure S6](#_Toc190954552) 10**

**[Figure S7 1](#_Toc190954552)1**

**[Figure S8 1](#_Toc190954552)2**

**[Figure S9 1](#_Toc190954552)3**

**[Figure S10 1](#_Toc190954552)4**

**[Figure S11 1](#_Toc190954552)5**

**[Table S1](#_Toc190954552) 16**

**[Table S2](#_Toc190954552) 17**

**[Table S3](#_Toc190954552) 18**

**[Table S4](#_Toc190954552) 19**

**[Table S5](#_Toc190954552) 20**

**[Table S6](#_Toc190954552) 21**

**[Table S7](#_Toc190954552) 22**

**Table S8 ............................................................................................................................................................... 23**

**Table S9 ............................................................................................................................................................... 24**

**Table S10 ............................................................................................................................................................. 25**

**[Reference 2](#_Toc190954553)6**

# Section **I: FNAC samples digitization and reviewing**

Slides used for the study were obtained through ultrasound-guided fine needle aspiration Cytopathology (FNAC) procedure performed on patients with thyroid nodules who had not undergone systemic or local treatment before.

We conducted a retrospective study about 18,360 thyroid FNAC samples from three centers, Southern Medical University's Nan Fang Hospital (SMUH), Zhengzhou University First Affiliated Hospital (ZUFAH), and Peking University Shenzhen Hospital (PUSH), to train and test the AI-TFNA model. These thyroid FNAC slides were digitized to WSIs by three type of scanners, including the Hamamatsu NanoZoomer S360 (NZ), ShengQiang Scanim SQS-40P (SQS), and Leica Aperio GT450 (LA), at 20 or 40 magnifications (**Figure S2**). These WSIs were diagnosed on the TBSRTC guidelines by three senior cytopathologists and thyroid nodules were classified into six categories from TBS Ⅰ to TBS VI. TBS Ⅰ include the unsatisfied samples and the samples with a poor-scan-quality. If WSIs that could not reach a unanimous diagnosis, additional diagnostic materials from the same thyroid nodules were assessed, such as ultrasound result, cell blocks and conventional smears. A unanimous diagnosis from this review was accepted as the final label. For discrepant cases, the final diagnostic label tended to the label with the most votes. Samples with no majority agreement (i.e., all three cytopathologists provided differing diagnoses) were excluded from the model training dataset. Among the development of AI-TFNA’s cohort (4421WSI), there were 1395 TBS II (Benign nodules), 796 TBS V (Suspicious for malignancy) and 2078 TBS VI (Malignant) thyroid nodules for the development of AI-TFNA, in addition, 152 TBS Ⅰ were used to conduct a decision logic to AI-TFNA for the quality control of slides. For the specimens with unclear diagnostic significance (TBS III) and follicular tumors (TBS IV) with unclear cytopathological significance regarding capsule and vascular invasion, due to the difficulty in making a definitive diagnosis through cytopathology and the small number of samples collected, we did not include them in the model development phase. After the model was built, we used TBS III and TBS IV samples for testing directly.

In the external validation datasets (2,016 samples for the external validation of AI-TFNA and 137 samples for the TBS III and TBS IV evaluation), thyroid FNAC slides were digitized by PRECICE 600 scanner, KF-PRO-400-HI and SQS-600P in Fujian Cancer Hospital (FCH), West China Hospital, Sichuan University (WCH), and Affiliated Hospital of Nantong University (AHNU), and stored in TMAP, kfb, sdpc, at 40 magnifications. Huayin Health Medical Group (HHMG) was digitized by SQS-600P at 20 magnifications (**Figure S2C-2D**).

BRAF-V600E is one of the most important gene in the diagnosis of benign and malignant thyroid nodules, and TBSRTC recommends the molecular detection of TBS III, IV and V in order to increase the accuracy of thyroid nodules diagnosis in clinical work^1, 2^. We collected the genetic results of 290 patients (290 WSI) about BRAF-V600E from SMUH to train the gene prediction module. The detection samples of BRAF-V600E were obtained from paraffin sections of the corresponding thyroid biopsy of the FNAC slides or the paraffin sections of the same nodule after surgery. Thyroid cancer BRAF gene V600E mutation detection kit was used for detection by fluorescence PCR. Mutations in the BRAF-V600E gene were found in 211 of the samples.

# Section **II: Datasets annotation and reviewing**

2,600 annotated image patches were obtained for the development of nucleus segmentation module, 2,080 image patches used for training, 260 patches used for validation and 260 patches used for testing. In the cell classification module, 291,502 ROIs were obtained, 256,858 ROIs were used for training and 34,644 validations. These data were obtained in three ways and shown in **Figure S2B**: two annotation ways included manual annotation and semi annotation via YOLOv5 & SEG-DETECT; and then we used color normalization to extend datasets. The corresponding class was then assigned to ROIs. The annotation team contained five members, three junior cytopathologists manual annotated data and two senior cytopathologists conducted the review. They used an opensource Artificial Intelligence-Pathology Annotation software (AI-PathA, <https://mark118.ai.pathologycn.com:6046/)> for manual and semi-supervised labelling process.

1. **Manual annotations:** This way were consisted with two parts: (**a)** **Nucleus segmentation**: cytopathologists annotated the boundary of each nucleus in sliding-patch images with 2048×2048 resolution in AI-PathA. (**b)** **Cell type classification**: Cytopathologists used AI-PathA to annotate the bounding box of each cell or cell cluster in WSI according to TBSRTC diagnostic criteria, meanwhile, they assigned classification labels (C1~C15) to each annotation area. After the primary cytopathologists finished their work, the annotations were further reviewed by senior cytopathologists in order to acquire an accurate data set.
2. **Semi-supervised annotation**: This part included YOLOv5 detection module and SEG-DETECT. A multi-classification YOLOv5 detection module was trained on manually annotated data to accelerate the speed of data annotation, leveraging its high recall rate and efficiency^3, 4^. The module initially detected lesion classification and generated rectangular regions of interest for subsequent semi-automatic labeling processes. SEG-DETECT is one of the main models of our pipeline and will be introduced in detail later. SEG-DETECT also generated ROI regions for doctors to screen at this step, which was a part of semi-supervised annotation. Any false ROI in the inference results was corrected by cytopathologist using AI-PathA, and the cropped images for each ROI were annotated to different defined categories containing relevant information. Finally, the detected images were further evaluated by two senior cytologists to rule out any non-targeted ROI. They annotated 17 class in this way.
3. **Color normalization:** For dataset augmentation, every 20% of the data in C1~C13 cell classification were processed to generate new training data by the Vahadane color normalization algorithm^5^. The Vahadane color normalization algorithm uses a Sparsity Non-negative Matrix Factorization method to capture the biological principle of discreteness of biological structures, and then combines the density map of source image with color appearance of the target image to generate the structure-preserving normalized source image.

**Section III : Image Appearance Migration (IAM)**

In the process of IAM, we used 3612 WSI with BD pap from SMUH as the reference dataset, 411 WSI with TP pap from ZUFAH, 428 WSI with BD HE from PUSH, 188 WSI with TP pap from FCH, 180 WSI with BD pap from WCH, and 210 WSI with BD pap from AHNU as the source dataset. In addition, during our experiments, it was found that the staining of HHMG was highly consistent with the reference dataset, so we did not perform IAM on this dataset (**Figure S10A**).

To further enhance generalizability and evaluate the performance of AI-TFNA on samples from different institutes, we employed the Image Appearance Migration (IAM) for validation. The SMUH dataset, which was developed under, was used as the reference dataset. This approach ensures that the validation reflects real-world scenarios, allowing us to assess the robustness and reliability of the AI-TFNA model when applied to varying sample conditions. The pseudo code of conduct the IAM process was shown next **Table S10**, the details and testing results were shown in **Figure S9** **- S11**.

**Section Ⅳ: Statistical Analysis**

For nucleus segmentation tasks, metrics like Dice score, intersection over union (IoU), mean IoU (mIoU), F1-score, and pixel accuracy are employed to measure the accuracy and effectiveness of the segmentation module in precisely delineating cell nuclei boundaries. We used recall and precision to evaluate the performance of the SEG-DETECT module. In cell classification tasks, evaluation metrics such as accuracy-top1, accuracy-top5, precision, and recall are used to assess the model's performance in accurately classifying images. Moreover, in the context of WSI-level classification, the module's performance was assessed through the calculation of precision, recall, F1-score, accuracy and analysis of the confusion matrix. As for the validation of AI-TFNA, the model's performance was assessed by Sensitivity, Specificity, Positive Predictive Value (PPV), Negative Predictive Value (NPV), Accuracy and the analysis of the confusion matrix. In addition, we computed 95%CI for all performance metrics to provide a precise measure. The diagnosis time of cytopathologists with and without AI-TFNA assistance was compared using a two-tailed paired t-test and the Wilcoxon signed-rank test. Statistical significance was defined as a p-value ≤ 0.05, and all analyses were performed using IBM SPSS Statistics 26. AI-TFNA is a multi-class model. For the generalization validation, we utilized several metrics for each category—including sensitivity, specificity, PPV, and NPV—as well as the average of these metrics across all categories. The important formulas were as follows (TP: True Positive; FP: False Positive; FN: False Negative; TN: True Negative):

1. $Precision=\frac{\mathrm{TP}}{TP+FP}$
2. $Recall=\frac{\mathrm{TP}}{TP+FN}$
3. $F1-score=2*\frac{Precision*Recall}{Precision+Recall}$
4. $\mathrm{Accuracy}=\frac{\mathrm{TP}+TN}{TP+FP+FN+TN}$
5. $\mathrm{Sensitivity}=\frac{\mathrm{TP}}{TP+FN}$
6. $\mathrm{Specifity}=\frac{TN}{TN+FP}$
7. $\mathrm{PPV}=\frac{\mathrm{TP}}{TP+FP}$
8. $\mathrm{NPV}=\frac{TN}{\mathrm{FN}+\mathrm{TN}}$


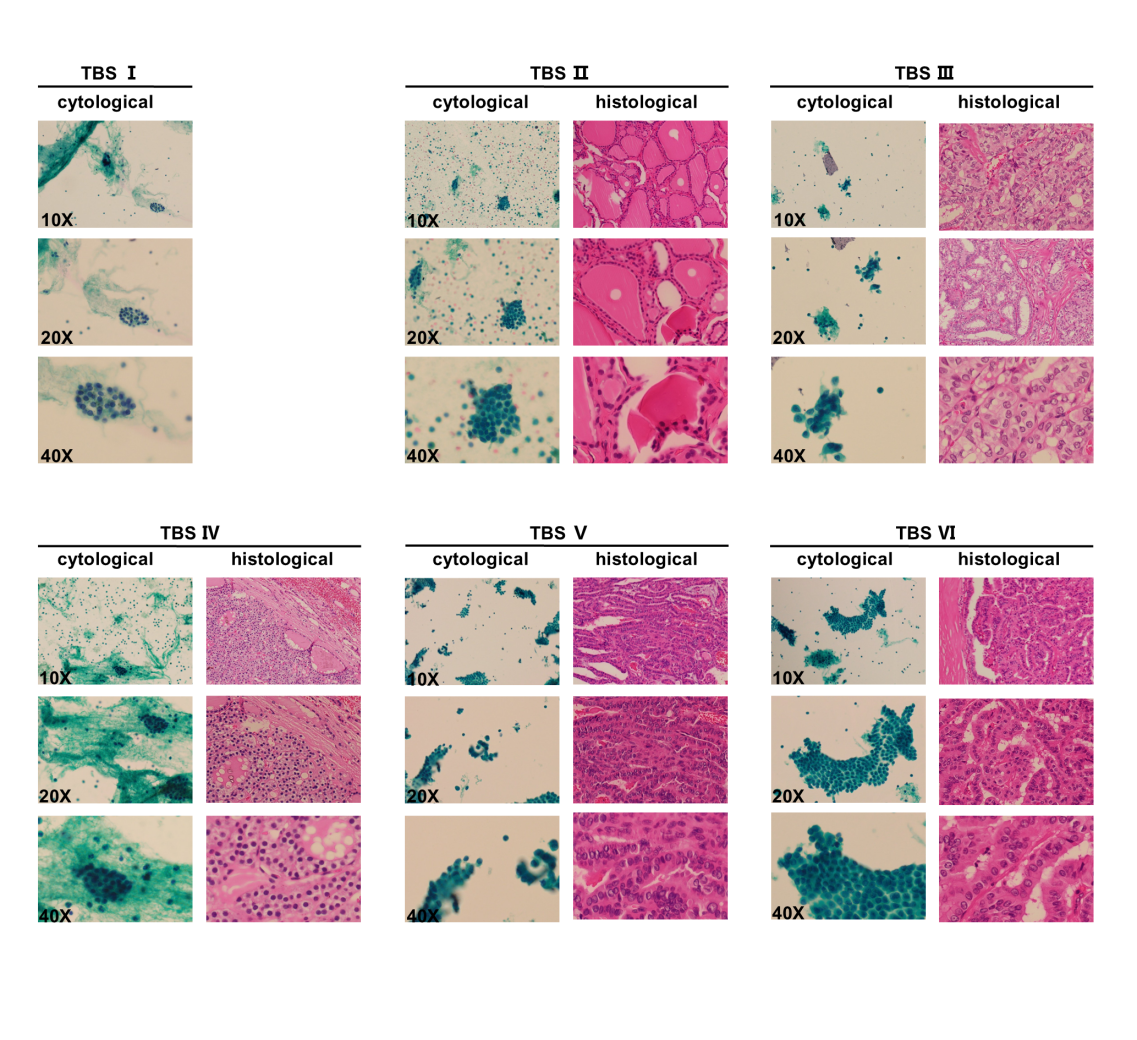


**Figure S1 The Bethesda System for Reporting Thyroid Cytopathology (TBSRTC) with main cytological features and the corresponding histopathology images.**TBSRTC includes six diagnostic categories: (I) nondiagnostic: less than six thyroid follicular cell clusters, with less than 10 cells per cluster; improper preparation, poor staining, or occluded follicular cells; only cystic fluid or blood. (II) Benign: sparse or moderate number of follicular cells in the specimen; monolayer follicular cells were spaced in a honeycomb pattern no nuclear enlargement or cell atypia; (III) Atypia of undetermined significance (AUS): most of the follicular epithelial cells were arranged in benign follicular structures, and a few had mild irregularity of nuclear membrane; the nucleus had grooves but lacked intranuclear pseudoinclusions. (IV) Follicular Neoplasm (FN): medium or abundant cells in the sample; the morphology of the cells was simple and the cytoplasm was abundant; the cells were crowded in clusters or microfollicles. (V) Suspicious for Malignancy (SFM): the characteristics of malignant tumor (nuclear crowding, nuclear staining, irregularity of nuclear membrane, grooves, etc.), but it was not enough to make a definite diagnosis. (VI) Malignant: the follicular epithelial cells were arranged in papillary and/or lamellar monolayers; crowded or overlapping nuclei, grooves, pseudoinclusion, pale nuclei, single or multiple small nucleoli; multinucleated giant cells. These images show the main features from TBS I to TBS VI and their corresponding histopathology images. upper 10X, middle 20X, lower 40X.

**
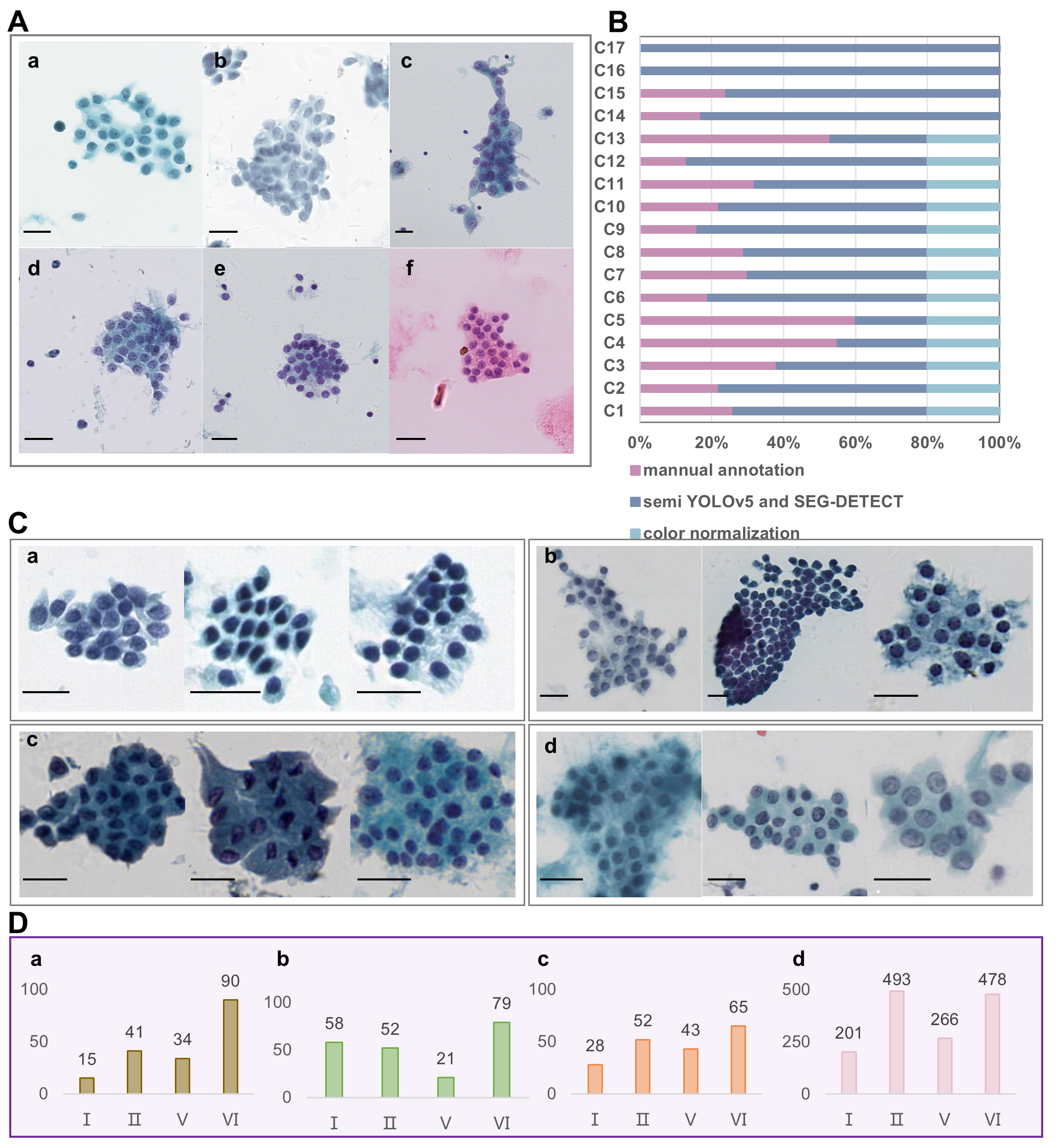
**

**Figure S2 Samples digitalization and acquisition of datasets. (A)** FNAC slides from different institutions were scanned using different scanners to form WSI (Scale Bars:20μm). **a** SMUH, Malignant, BD Pap, Scanim SQS 40X. **b** SMUH, Malignant, BD Pap, LA 40X. **c** ZUFAH, Malignant, TP Pap, NZ 40X. **d** SMUH, Benign, BD Pap, NZ 40X. **e** SMUH, Benign, TP Pap, NZ 40X. **f** PUSH, Benign, BD HE, NZ 40X. (**B)** Cell annotation data were obtained in three ways: manual annotation by cytopathologists, semi YOLOv5 and SEG-DETECT for semi-supervised labeling, and color normalization for data augmentation. (**C)** **a** WCH opted for BD Pap with KF-PRO-400-HI scanner. **b** AHNU opted for BD Pap with SQS-600P. **c** FCH opted for TP Pap with PRECICE 600 scanner. **d** HHMG opted for BD Pap with SQS-600P. Scale Bars:20μm. (**D)** 2,016 samples from WCH (**a**), AHNU (**b**), FCH (**c**) and HHMG (**d**) for the external validation. FNAC: Fine Needle Aspiration Cytology; WSI: Whole-Slide Image; SUMH: Southern Medical University's Nan Fang Hospital; BD Pap: BD SurePath system with Papanicolaou staining; SQS: ShengQiang Scanim; LA: Leica Aperio; ZUFAH: Zhengzhou University First Affiliated Hospital; TP: ThinPrep with Pap stain; NZ: Hamamatsu NanoZoomer; PUSH: Peking University Shenzhen Hospital; BD HE: BD SurePath with Hematoxylin and Eosin stain; FCH: Fujian Cancer Hospital; WCH: West China Hospital, Sichuan University; AHNU: Affiliated Hospital of Nantong University; HHMG: Huayin Health Medical Group.


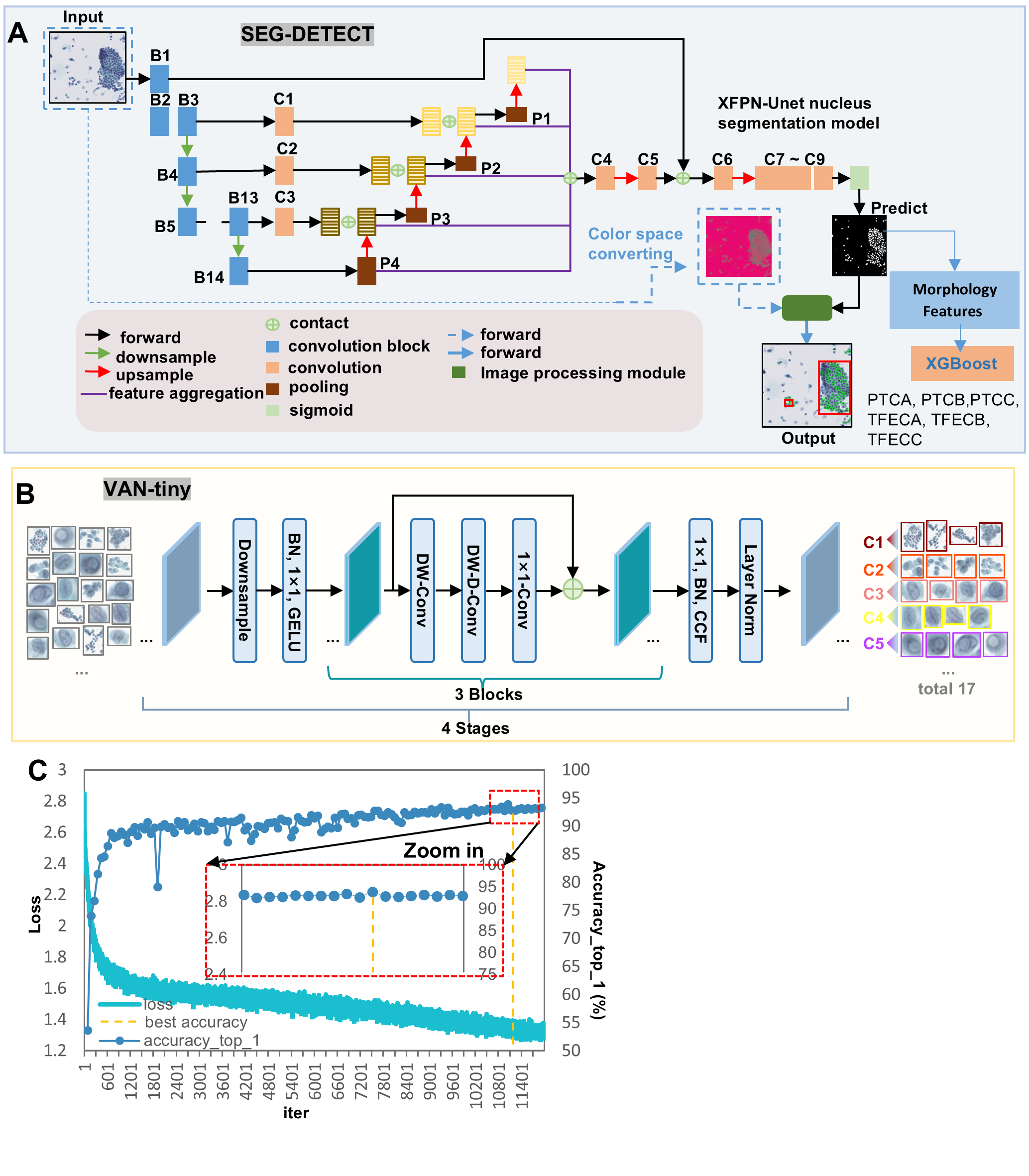


**Figure S3** **The details in SEG-DETECT module and VAN-tiny module.** (**A).** SEG-DETECT module includes four parts: XFPN-U-Net module for the nuclear segmentation, the color space transformation module for eliminating some nucleus-similar debris, the image post-processing module for more precision detection and the XGBoost cell classification module with morphological feature extraction. **(B)**. The VAN-tiny module was constructed by four Visual Attention Network stages with three Large Kernel Attention blockes in each stage to perform a seventeen-catelogies cell images classification task. **(C).** The loss curve of VAN-tiny module showed that the precision reached a maximum of 93.95% when training 10,922 iterations (about 227 epochs).

| 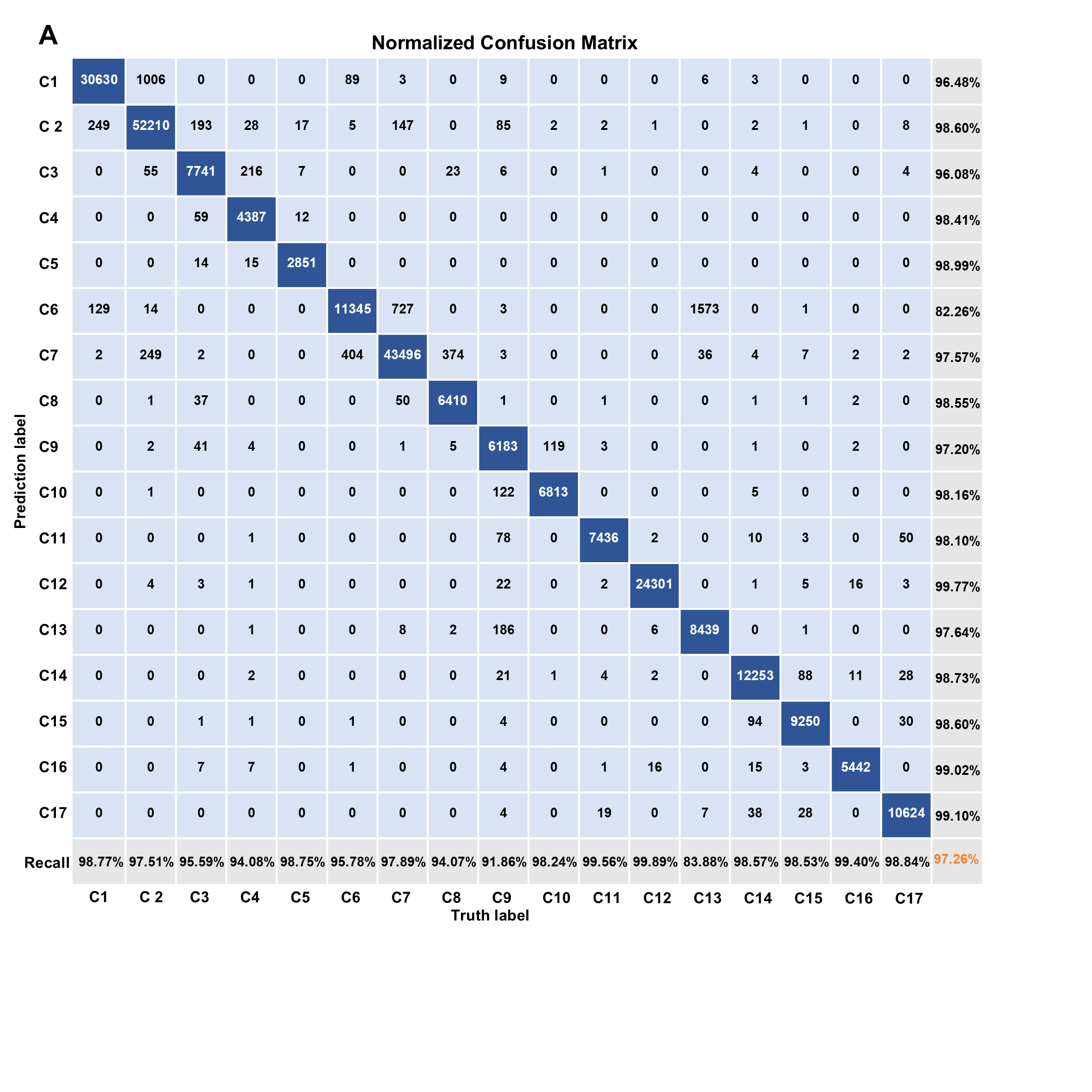 |  |
| --- | --- |
| 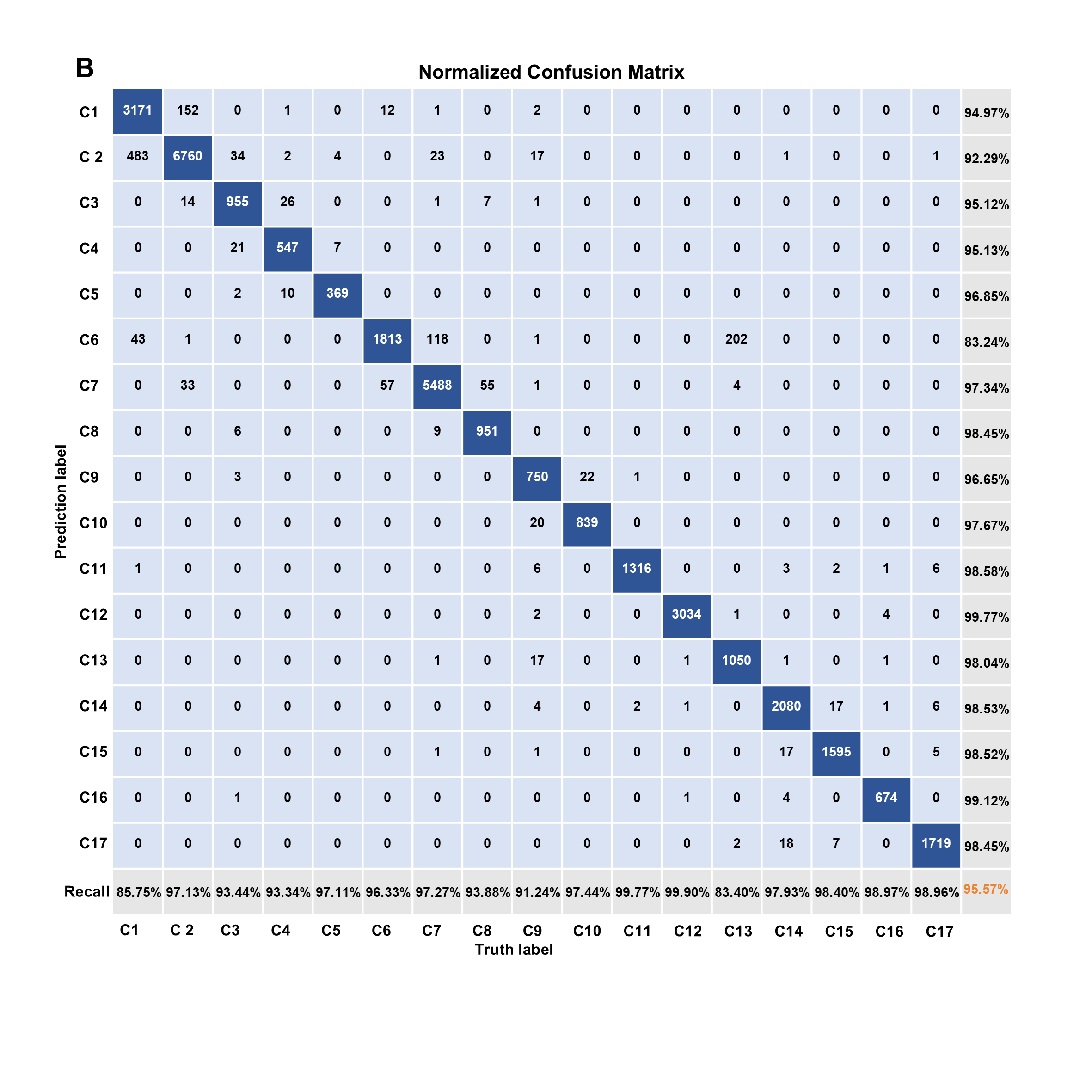 | 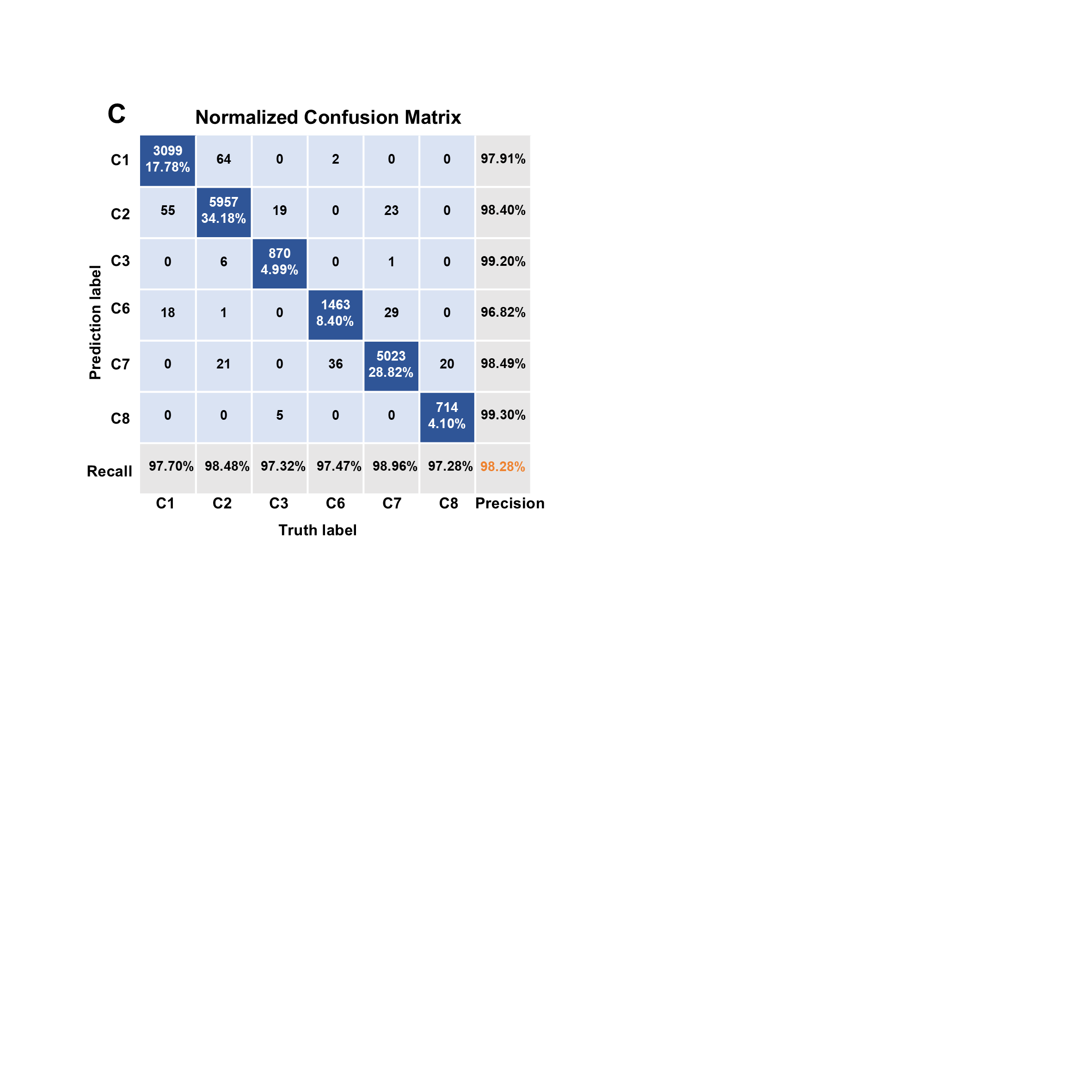 |

**Figure S4 The performance of VAN-tiny in training and validation set. (A).** The confusion matrix of the VAN-tiny in the training set in 17 class. (**B).** The confusion matrix of the VAN-tiny in the validation set in 17 class. (**C).** VAN-tiny C1~C3 and C6~C8 cells consistent with cell XGBoost diagnosis to improve the accuracy of cell classification models in the validation set.


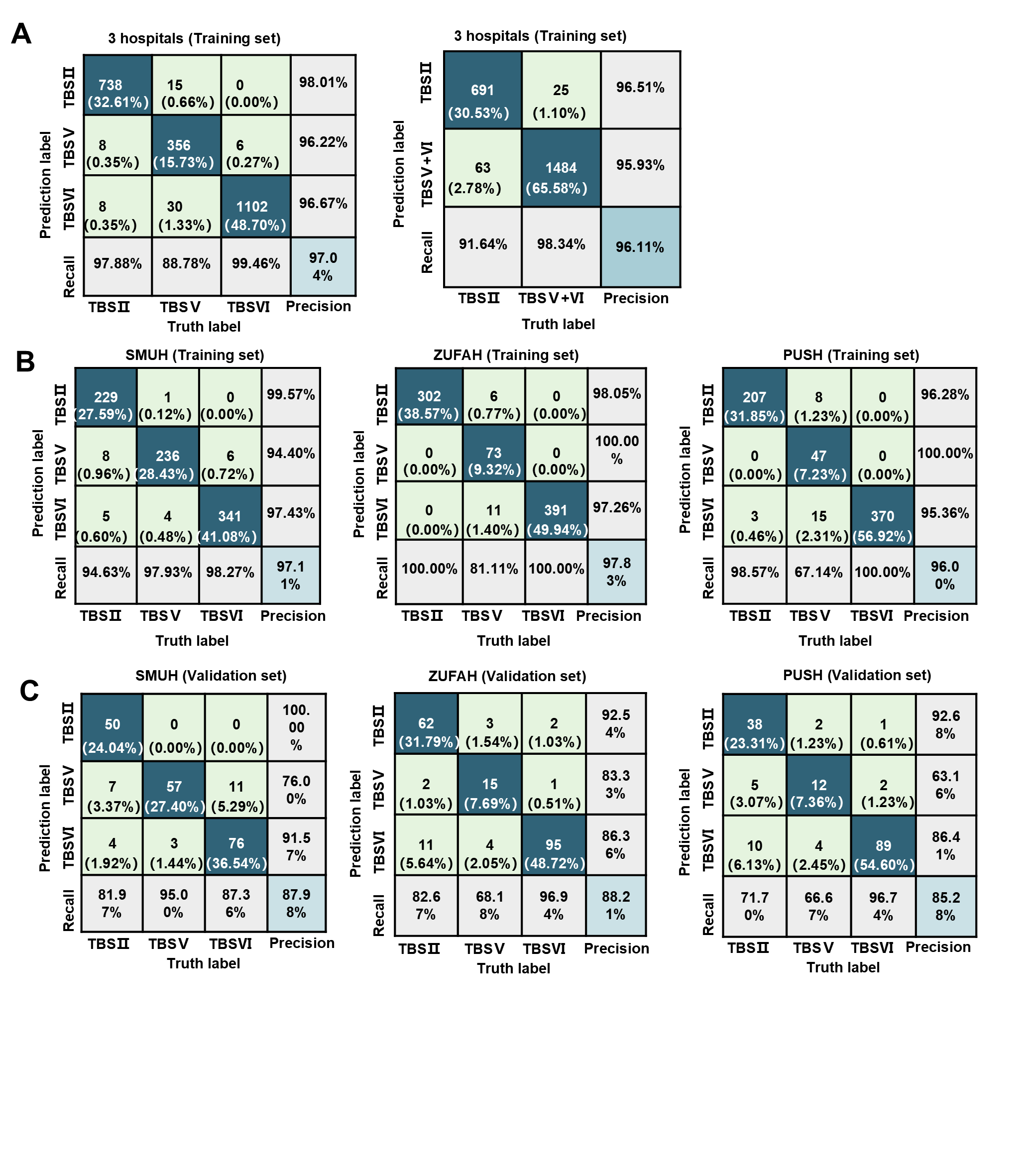


**Figure S5** **The performance of WSI-level XGBoost in training and validation set. (A).** The confusion matrix of WSI XGBoost in the training set with three classifications (TBS Ⅱ/TBS Ⅴ/TBS Ⅵ) and two classifications (TBS Ⅱ/TBS Ⅴ+Ⅵ) in SMUH, ZUFAH and PUSH. (**B).** The confusion matrix of WSI XGBoost module in the training set of SMUH, ZUFAH and PUSH with three classifications (TBS Ⅱ/TBS Ⅴ/TBS Ⅵ). (**C).** The confusion matrix of WSI XGBoost model in the validation set of SMUH, ZUFAH and PUSH with three classifications (TBS Ⅱ/TBS Ⅴ/TBS Ⅵ). SMUH: Southern Medical University's Nan Fang Hospital; ZUFAH: Zhengzhou University First Affiliated Hospital; PUSH: Peking University Shenzhen Hospital.


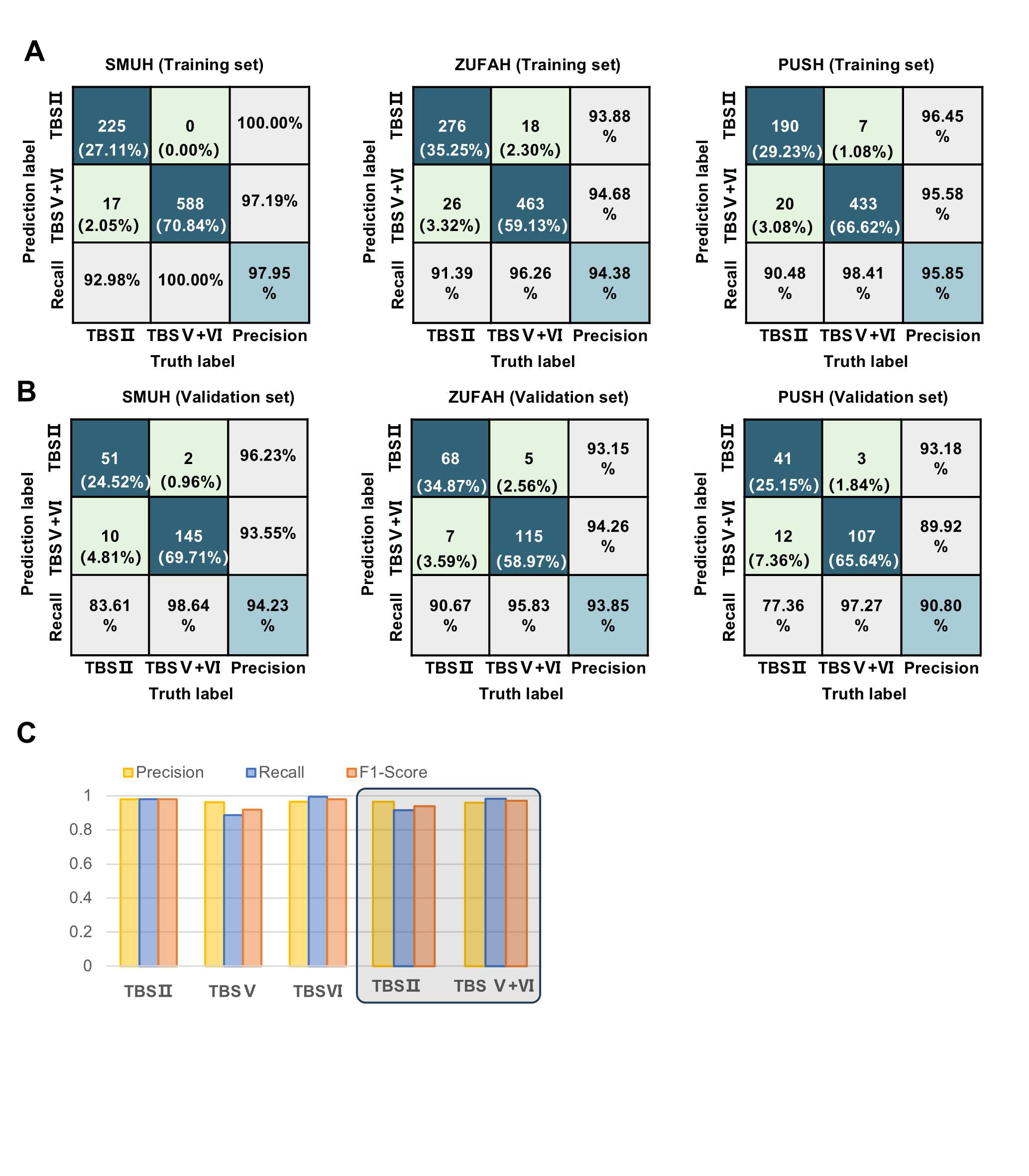


**Figure S6 The performance of WSI-level XGBoost in the training and validation set.** (**A).** The confusion matrix of WSI XGBoost module in the training set of SMUH, ZUFAH and PUSH with two classifications (TBS Ⅱ/TBS Ⅴ+Ⅵ). (**B).** The confusion matrix of WSI XGBoost module in the validation set of SMUH, ZUFAH, PUSH with two classifications (TBS Ⅱ/TBS Ⅴ+Ⅵ). (**C).** The precision, recall and F1-Score in XGBoost for three-class classification (TBS Ⅱ/TBS Ⅴ/TBS Ⅵ) and two classification (TBS Ⅱ/TBS Ⅴ+Ⅵ) in the training set. SMUH: Southern Medical University's Nan Fang Hospital; ZUFAH: Zhengzhou University First Affiliated Hospital; PUSH: Peking University Shenzhen Hospital.


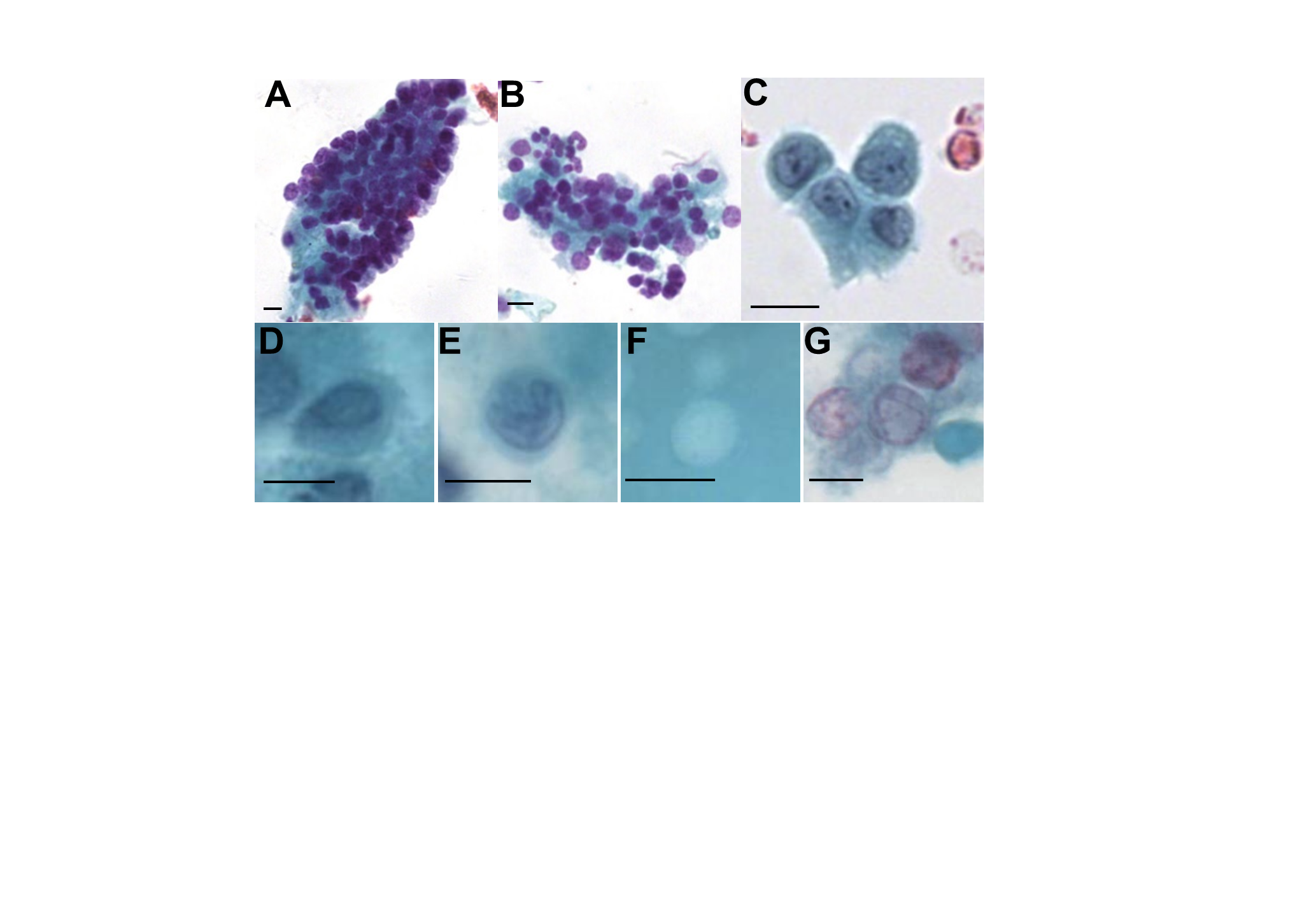


**Figure S7 The difficulty in the cell classification module (Scale Bars:10μm).** (**A).** Thyroid follicular epithelial cells with papillary hyperplasia were classified as PTCA. (**B).** A large number of aggregated lymphocytes in ThinPrep mixed with individual follicular epithelial cells were classified as TFECA. (**C).** The macrophages were classified as PTC. (**D).** When the color difference between the nucleus and cytoplasm was small, the nuclear membrane could be easily identified as grooves. (**E).** The irregular cell nucleus of neutrophils were easily identified as grooves. (**F).** Bubbles in the slides were classified as pseudoinclusion. (**G).** Structures resembling pseudoinclusion in nonthyroid follicular epithelial cells.


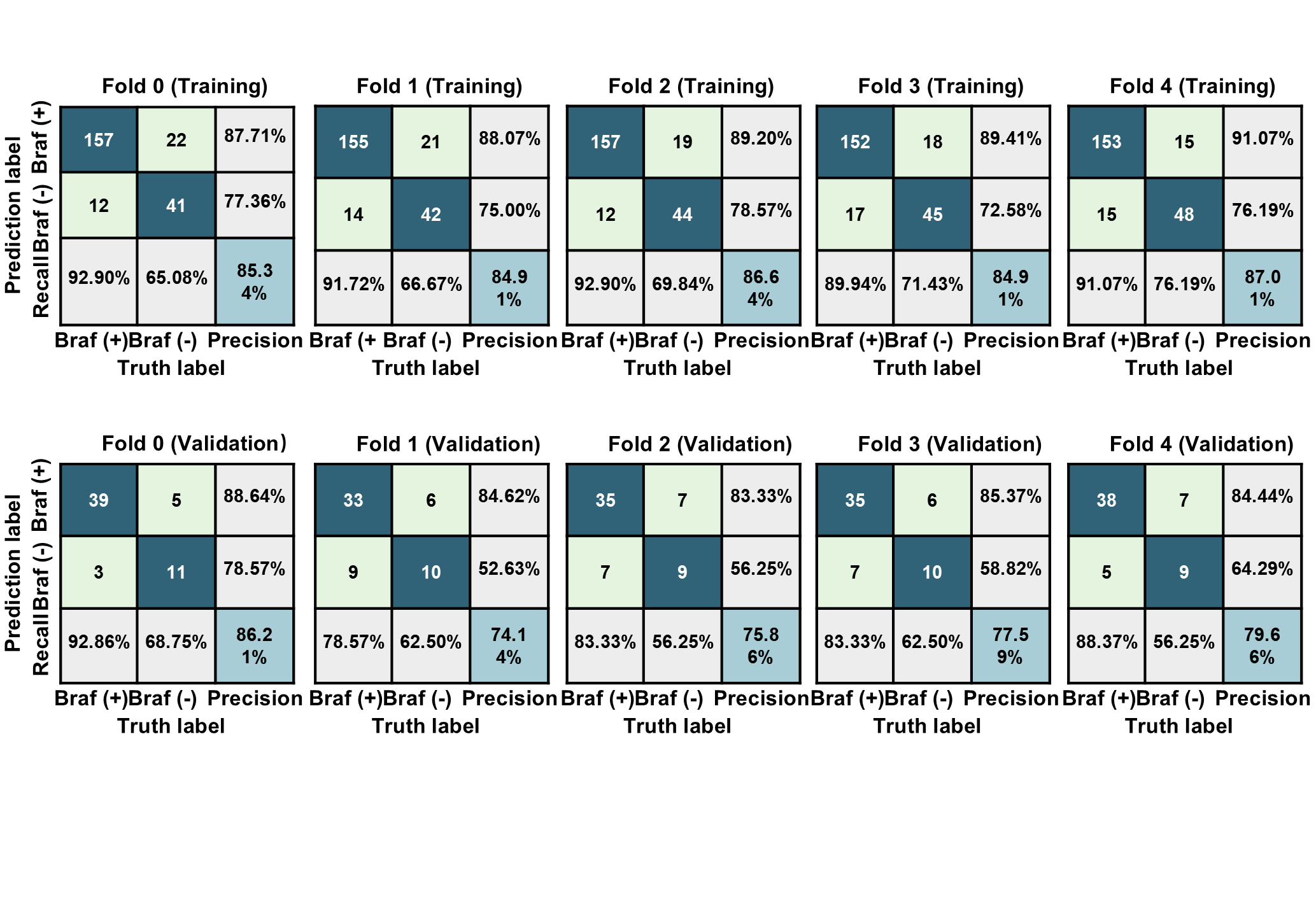


**Figure S8 The confusion matrix of Gene-XGBoost module in the training and validation set.** The picture were the results of fivefold cross-validated from Fold 0 to Fold 4. Upper: training set; lower: validation set.


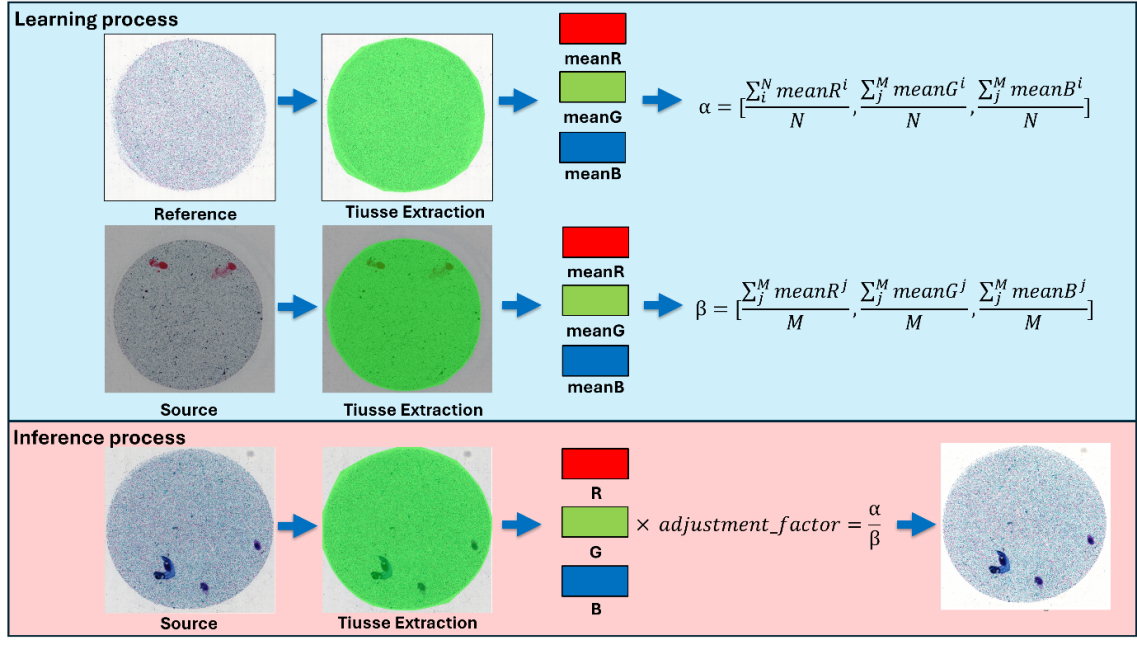


**Figure S9 The learning and inference processes for tissue extraction and image Appearance Migration (IAM) in image analysis.** The learning process involves calculating mean values (meanR, meanG, meanB) from a reference image and applying these values to adjust the source image to match the reference. The adjustment factor α is determined by the ratios of the mean values. During the inference process, the adjusted source image is used to generate a final output image with migrated colors and styles.

**
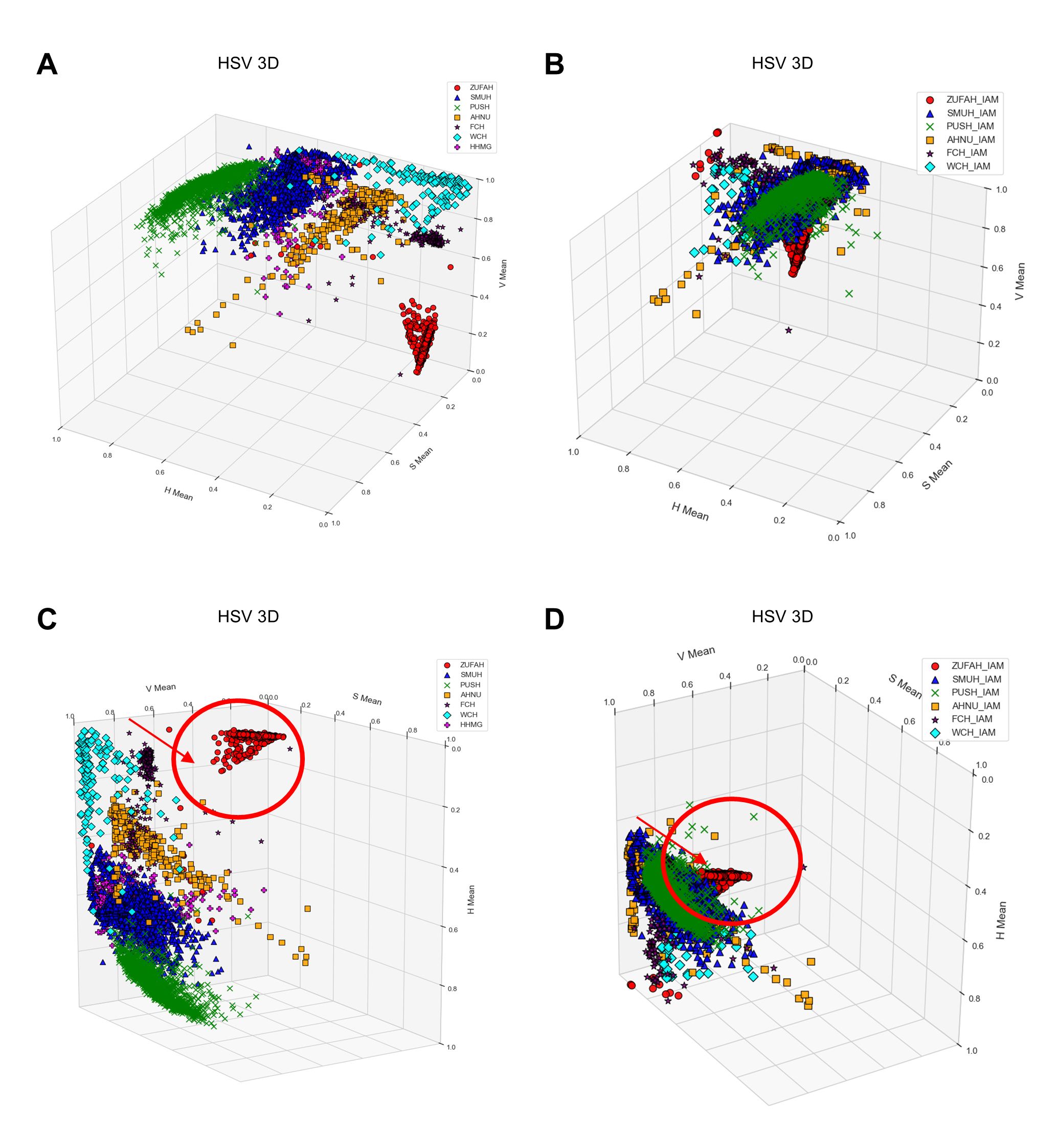
**

**Figure S10 Differences in HSV spatial distribution between raw data (A) and data after IAM (B)**. The HSV (Hue, Saturation, Value) color space aligns more closely with human visual perception, as it separates color, saturation, and brightness components. Researchers commonly use HSV color distribution to highlight salient regions in images. In panels (A) and (B), changes in color distribution across different hospitals can be observed. Panels (C) and (D) provide an alternative view of (A) and (B), respectively. For instance, the red circles in (C) and (D) indicate that the HSV distributions of samples from ZUFAN have been transformed and clustered. This demonstrates that after applying IAM, the color values in the dataset tend to cluster in HSV space, thereby reducing the variation in color distribution present in the original dataset.

**
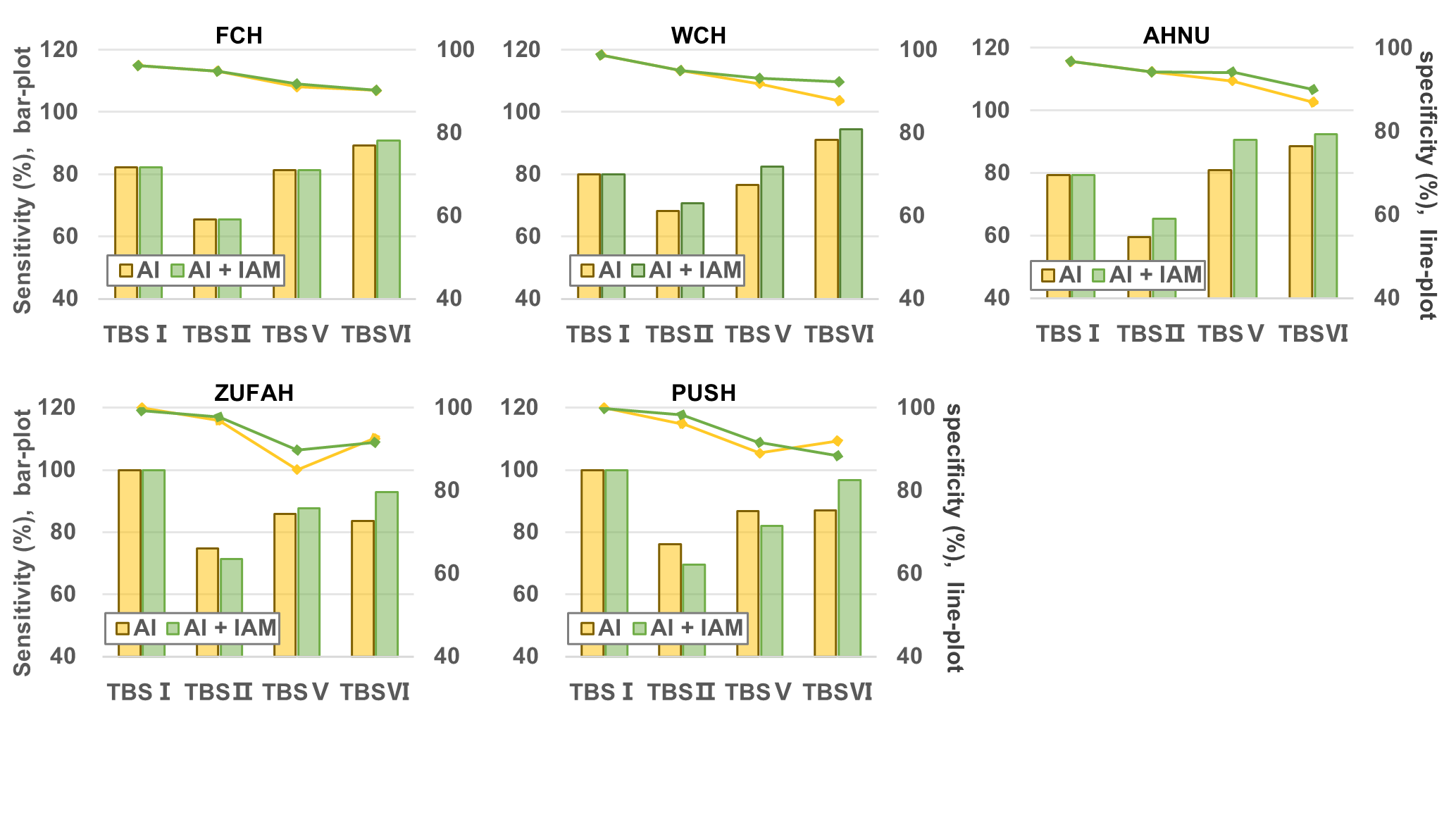
**

**Figure S11 The evulation of Image Appearance Migration (IAM).** The IAM technique to improve the generalizability of AI-TFNA.,the results in FCH, WCH, AHNU, ZUFAH, and PUSH, respectively.

**Table S1 The classification and definition of annotated categories.**

|  | Annotation classification | Nature | Category | Definition |
| --- | --- | --- | --- | --- |
| C1 | PTCA | Malignant | Thyroid follicular cells | Thyroid follicular epithelial cells with atypia are arranged in clumps (cells number≥10) |
| C2 | PTCB | Malignant | Thyroid follicular cells | Thyroid follicular epithelial cells with atypia are arranged in clumps(2≤cells number ＜10) |
| C3 | PTCC | Malignant | Thyroid follicular cells | A single scattered thyroid follicular epithelial cell with atypia |
| C4 | NUCLE | Malignant | Thyroid follicular cells | Grooves |
| C5 | INCIS | Malignant | Thyroid follicular cells | Pseudo-inclusions |
| C6 | TFECA | Benign | Thyroid follicular cells | Thyroid follicular epithelial cells without atypia are arranged in clumps (cells number≥10) |
| C7 | TFECB | Benign | Thyroid follicular cells | Thyroid follicular epithelial cells without atypia are arranged in clumps(2≤cells number ＜10) |
| C8 | TFECC | Benign | Thyroid follicular cells | A single scattered thyroid follicular epithelial cell without atypia |
| C9 | MOA | Benign | Histocyte | Macrophages |
| C10 | MOFE | Benign | Histocyte | Hemosiderin cells |
| C11 | COLLOID | Benign | Normal component | Thyroid colloids |
| C12 | NC | Benign | Inflammatory cell | Inflammatory cell |
| C13 | NCA | Benign | Inflammatory cell | Inflammatory cell in clusters |
| C14 | RUBBB | Trash | Trash | Contaminated impurities, gunk, red blood cells, etc |
| C15 | RUBBR | Trash | Trash |  |
| C16 | RUBBQ | Trash | Trash |  |
| C17 | RUBBA | Trash | Trash |  |

**Table S2 Features of XGBoost module for the classification of cells.**

| **Methods** | Feature id | Annotation |
| --- | --- | --- |
| **SEG-detect** | NUM-NUCLEAR | The number of nuclear in cell |
| **SEG-detect** | AVG-AREA-NUCLEAR | The average nuclear area of cell |
| **SEG-detect** | AVG-PET-NUCLEAR | The average nuclear perimeter of cell |
| **SEG-detect** | AVG-MAXAL-NUCLEAR | The average nuclear major axis length of cell |
| **SEG-detect** | AVG-MINAL-NUCLEAR | The average nuclear minor axis length of cell |
| **SEG-detect** | AVG-ECC-NUCLEAR | The average nuclear eccentricity of cell |
| **SEG-detect** | AVG-SOL-NUCLEAR | The average nuclear solidity of cell |
| **SEG-detect** | AVG-SF-NUCLEAR | The average nuclear shape factor of cell |
| **SEG-detect** | AVG-CA-NUCLEAR | The average nuclear convex area of cell |
| **SEG-detect** | AVG-ED-NUCLEAR | The average nuclear equivalent diameter of cell |
| **SEG-detect** | MEDI-AREA-NUCLEAR | The median nuclear area of cell |
| **SEG-detect** | MEDI-PET-NUCLEAR | The median nuclear perimeter of cell |
| **SEG-detect** | MEDI-MAXAL-NUCLEAR | The median nuclear major axis length of cell |
| **SEG-detect** | MEDI-MINAL-NUCLEAR | The median nuclear minor axis length of cell |
| **SEG-detect** | MEDI-ECC-NUCLEAR | The median nuclear eccentricity of cell |
| **SEG-detect** | MEDI-SOL-NUCLEAR | The median nuclear solidity of cell |
| **SEG-detect** | MEDI-SF-NUCLEAR | The median nuclear shape factor of cell |
| **SEG-detect** | MEDI-CA-NUCLEAR | The median nuclear convex area of cell |
| **SEG-detect** | MEDI-ED-NUCLEAR | The median nuclear equivalent diameter of cell |

**Table S3 Features of XGBoost module for the diagnosis of thyroid FNAC WSI.**

| **Feature ID** |  |  |  | |  |  |
| --- | --- | --- | --- | --- | --- | --- |
| **SEG-detect** | **VAN-tiny** | **VAN-tiny** | **VAN-tiny** | | **VAN-tiny** | **VAN-tiny** |
| AVG-SC-PTCA | AVG-AREA-NUCLEAR-PTCA | AVG-ED-NUCLEAR-PTCB | AVG-SF-NUCLEAR-TFECA | | AVG-ECC-NUCLEAR-TFECC | AVG-P-TFECA |
| AVG-SC-PTCB | AVG-PET-NUCLEAR-PTCA | AVG-NUM-NUCLEAR-PTCB | AVG-CA-NUCLEAR-TFECA | | AVG-SOL-NUCLEAR-TFECC | AVG-P-TFECB |
| AVG-SC-PTCC | AVG-MAXAL-NUCLEAR-PTCA | AVG-AREA-NUCLEAR-PTCC | AVG-ED-NUCLEAR-TFECA | | AVG-SF-NUCLEAR-TFECC | AVG-P-TFECC |
| AVG-SC-NUCLEAR | AVG-MINAL-NUCLEAR-PTCA | AVG-PET-NUCLEAR-PTCC | AVG-NUM-NUCLEAR-TFECA | | AVG-CA-NUCLEAR-TFECC | AVG-P-NUCLEAR |
| AVG-SC-INCIS | AVG-ECC-NUCLEAR-PTCA | AVG-MAXAL-NUCLEAR-PTCC | AVG-AREA-NUCLEAR-TFECB | | AVG-ED-NUCLEAR-TFECC | AVG-P-INCIS |
| AVG-SC-TFECA | AVG-SOL-NUCLEAR-PTCA | AVG-MINAL-NUCLEAR-PTCC | AVG-PET-NUCLEAR-TFECB | | AVG-NUM-NUCLEAR-TFECC | AVG-P-OTHERS |
| AVG-SC-TFECB | AVG-SF-NUCLEAR-PTCA | AVG-ECC-NUCLEAR-PTCC | AVG-MAXAL-NUCLEAR-TFECB | | N-PTCA | MEDI-P-PTCA |
| AVG-SC-TFECC | AVG-CA-NUCLEAR-PTCA | AVG-SOL-NUCLEAR-PTCC | AVG-MINAL-NUCLEAR-TFECB | | N-PTCB | MEDI-P-PTCB |
| AVG-SC-OTHER | AVG-ED-NUCLEAR-PTCA | AVG-SF-NUCLEAR-PTCC | AVG-ECC-NUCLEAR-TFECB | | N-PTCC | MEDI-P-PTCC |
| MEDI-SC-PTCA | AVG-NUM-NUCLEAR-PTCA | AVG-CA-NUCLEAR-PTCC | AVG-SOL-NUCLEAR-TFECB | | N-TFECA | MEDI-P-TFECA |
| MEDI-SC-PTCB | AVG-AREA-NUCLEAR-PTCB | AVG-ED-NUCLEAR-PTCC | AVG-SF-NUCLEAR-TFECB | | N-TFECB | MEDI-P-TFECB |
| MEDI-SC-PTCC | AVG-PET-NUCLEAR-PTCB | AVG-NUM-NUCLEAR-PTCC | AVG-CA-NUCLEAR-TFECB | | N-TFECC | MEDI-P-TFECC |
| MEDI-SC-NUCLEAER | AVG-MAXAL-NUCLEAR-PTCB | AVG-AREA-NUCLEAR-TFECA | AVG-ED-NUCLEAR-TFECB | | N-NUCLEAR | MEDI-P-NUCLEAR |
| MEDI-SC-INCIS | AVG-MINAL-NUCLEAR-PTCB | AVG-PET-NUCLEAR-TFECA | AVG-NUM-NUCLEAR-TFECB | | N-INCIS | MEDI-P-INCIS |
| MEDI-SC-TFECA | AVG-ECC-NUCLEAR-PTCB | AVG-MAXAL-NUCLEAR-TFECA | AVG-AREA-NUCLEAR-TFECC | | N-OTHERS | MEDI-P-OTHERS |
| MEDI-SC-TFECB | AVG-SOL-NUCLEAR-PTCB | AVG-MINAL-NUCLEAR-TFECA | AVG-PET-NUCLEAR-TFECC | | AVG-P-PTCA |  |
| MEDI-SC-TFECC | AVG-SF-NUCLEAR-PTCB | AVG-ECC-NUCLEAR-TFECA | AVG-MAXAL-NUCLEAR-TFECC | | AVG-P-PTCB |  |
| MEDI-SC-OTHER | AVG-CA-NUCLEAR-PTCB | AVG-SOL-NUCLEAR-TFECA | AVG-MINAL-NUCLEAR-TFECC | | AVG-P-PTCC |  |
| ***AVG-SC: The average detection score.**  ***MEDI-SC: The median detection score.**  ***AVG-AREA-NUCLEAR:The average nuclear area.**  ***AVG-PET-NUCLEAR:The average nuclear perimeter.**  ***AVG-MAXAL-NUCLEAR:The average nuclear major axis length.**  ***AVG-MINAL-NUCLEAR:The average nuclear minor axis length.**  ***AVG-ECC-NUCLEAR:The average nuclear eccentricity.**  ***AVG-SOL-NUCLEAR:The average nuclear solidity.** | | | | *****AVG-SF-NUCLEAR:The average nuclear shape factor.  *****AVG-CA-NUCLEAR:The average nuclear convex area.  *****AVG-ED-NUCLEAR:The average nuclear equivalent diameter.  *****AVG-NUM-NUCLEAR:The average nuclear number.  *****N:The number of classes with the classifier prediction probability greater than 0.1  *****AVG-P:The average classification probability.  *****MEDI-P:The median classification probability. | | |

**Table S4 Accuracy of TBS I diagnosis.**

|  | Sensitivity  (95% CI) | Specificity  (95% CI) | PPV  (95% CI) | NPV  (95% CI) | Accuracy  (95% CI) |
| --- | --- | --- | --- | --- | --- |
| SMUH | 91.11%  (0.8862-0.9361) | 90.14%  (0.8752-0.9275) | 77.36%  (0.7369-0.8103) | 96.48%  (0.9487-0.9810) | 90.40%  (0.8782-0.9298) |
| ZUFAH | 73.33%  (0.6946-0.7721) | 96.99%  (0.9549-0.9848) | 90.00%  (0.8737-0.9263) | 90.77%  (0.8823-0.9331) | 90.60%  (0.8804-0.9298) |
| PUSH | 98.52%  (0.9746-0.9958) | 98.90%  (0.9799-0.9982) | 97.08%  (0.9560-0.9856) | 99.45%  (0.9802-0.9993) | 98.80%  (0.9785-0.9975) |
| Total | 87.65%  (0.8599-0.8932 ) | 95.34%  (0.9428-0.9641) | 87.44%  (0.8576-0.8912) | 95.43%  (0.9437-0.9649) | 93.27%  (0.9200-0.9453) |

SMUH: Southern Medical University's Nan Fang Hospital; ZUFAH: The Zhengzhou University First Affiliated Hospital; PUSH: Peking University Shenzhen Hospital.

**Table S5 The diagnostic performance of AI-TFNA alone, cytopathologists alone, and AI-TFNA-assisted cytopathologists.**

|  | Accuracy  (95% CI) | Sensitivity  (95% CI) | Specificity  (95% CI) | PPV  (95% CI) | NPV  (95% CI) |
| --- | --- | --- | --- | --- | --- |
| AI-TFNA | 75.17%  (0.6823-0.8211) | 84.10%  (0.7823-0.8997) | 91.22%  (0.8668-0.9576) | 82.59%  (0.7651-0.8868) | 90.85%  (0.8622-0.9548) |
| Cytopathologists without AI-TFNA | | | | | |
| Senior 1 | 89.93%  (0.8510-0.9476) | 89.53%  (0.8462-0.9445) | 96.36%  (0.9336-0.9937) | 88.54%  (0.8343-0.9366) | 96.71%  (0.9385-0.9958) |
| Senior 2 | 86.58%  (0.8110-0.9205) | 89.55%  (0.8463-0.9446) | 95.36%  (0.9198-0.9874) | 86.25%  (0.8073-0.9178) | 95.05%  (0.9157-0.9854) |
| Senior | 88.26%  (0.8460-0.9191) | 89.54%  (0.8606-0.9301) | 95.86%  (0.9360-0.9812) | 86.81%  (0.8297-0.9065) | 95.76%  (0.9347-0.9805) |
| Junior 1 | 72.48%  (0.6531-0.7965) | 70.95%  (0.6366-0.7824) | 89.88%  (0.8504-0.9472) | 78.36%  (0.7174-0.8497) | 90.14%  (0.8535-0.9493) |
| Junior 2 | 71.14%  (0.6387-0.7842) | 72.02%  (0.6482-0.7923) | 89.92%  (0.8509-0.9476) | 72.70%  (0.6554-0.7985) | 89.67%  (0.8479-0.9456) |
| Junior 3 | 77.85%  (0.7118-0.8452) | 81.10%  (0.7481-0.8738) | 92.06%  (0.8772-0.9640) | 79.36%  (0.7286-0.8586) | 91.85%  (0.8746-0.9624) |
| Junior | 73.83%  (0.6975-0.7790) | 74.69%  (0.7066-0.7872) | 90.62%  (0.8792-0.9332) | 76.35%  (0.7241-0.8029) | 90.52%  (0.8780-0.9323) |
| Cytopathologists with AI-TFNA | | | | | |
| Senior 1 | 89.26%  (0.8429-0.9423) | 89.38%  (0.8443-0.9432) | 95.88%  (0.9268-0.9907) | 90.98%  (0.8638-0.9558) | 96.36%  (0.9336-0.9937) |
| Senior 2 | 92.62%  (0.8842-0.9682) | 94.48%  (0.9081-0.9815) | 97.31%  (0.9471-0.9991) | 94.01%  (0.9020-0.9782) | 97.20%  (0.9455-0.9985) |
| Senior | 90.94%  (0.8460-0.9191) | 91.93%  (0.8883-0.9502) | 96.59%  (0.9453-0.9865) | 92.27%  (0.8924-0.9531) | 96.69%  (0.9466-0.9872) |
| Junior 1 | 77.85%  (0.7118-0.8452) | 78.68%  (0.7210-0.8526) | 91.59%  (0.8713-0.9604) | 83.77%  (0.7785-0.8969) | 91.79%  (0.8738-0.9620) |
| Junior 2 | 80.54%  (0.7418-0.8689) | 82.62%  (0.7653-0.8870) | 92.95%  (0.8884-0.9706) | 81.01%  (0.7471-0.8730) | 92.99%  (0.8888-0.9709) |
| Junior 3 | 86.58%  (0.8110-0.9205) | 87.40%  (0.8208-0.9273) | 95.11%  (0.9164-0.9857) | 88.42%  (0.8328-0.9356) | 95.10%  (0.9163-0.9857) |
| Junior | 81.66%  (0.7807-0.8524) | 82.90%  (0.7941-0.8639) | 93.22%  (0.9088-0.9555) | 83.90%  (0.8049-0.8731) | 93.26%  (0.9093-0.9558) |

Senior: the average of senior cytopathologists; Junior: the average of junior cytopathologists.

**Table S6 The diagnostic time of five pathologists and AI-TFNA.**

| Cytopathologists | Mean time (s) | P value |
| --- | --- | --- |
| Senior 1 | 148.48 | ＜0.001 |
| Senior 1 with AI-TFNA | 74.18 |  |
| Senior 2 | 150.89 | ＜0.001 |
| Senior 2 with AI-TFNA | 72.68 |  |
| Junior 1 | 168.45 | ＜0.001 |
| Junior 1 with AI-TFNA | 90.30 |  |
| Junior 2 | 204.13 | ＜0.001 |
| Junior 2 with AI-TFNA | 103.73 |  |
| Junior 3 | 184.79 | ＜0.001 |
| Junior 3 with AI-TFNA | 94.59 |  |
| AI-TFNA | 101.50 | - |

**Table S7 The result of cyto-histo evaluation in muilt-centers.**

| Hospitals | Number | Cyto.=Histo. | Cyto.≠Histo. | Accuracy  (95% CI) |
| --- | --- | --- | --- | --- |
| SMUH | 586 | 569 | 17 | 97.10%  (0.9574-0.9846) |
| ZUFAH | 475 | 442 | 33 | 93.05%  (0.9077-0.9534) |
| PUSH | 823 | 807 | 16 | 98.06%  (0.9711-0.9900) |
| Total | 1884 | 1818 | 66 | 96.50%  (0.9567-0.9733) |

SMUH: Southern Medical University's Nan Fang Hospital; ZUFAH: The Zhengzhou University First Affiliated Hospital; PUSH: Peking University Shenzhen Hospital.

**Table S8 The internal validation of AI-TFNA across different clinical settings.**

|  |  | Sensitivity  (95% CI) | Specificity  (95% CI) | PPV  (95% CI) | NPV  (95% CI) | Accuracy  (95% CI) |
| --- | --- | --- | --- | --- | --- | --- |
| Different stains & scanners | SMUH | 79.84%  (0.7859-0.8109) | 90.06%  (0.8913-0.9099) | 74.11%  (0.7274-0.7547) | 87.95%  (0.8693-0.8896) | 77.22%  (0.7591-0.7853) |
|  | ZUFAH | 80.67%  (0.7897-0.8237) | 90.21%  (0.8893-0.9149) | 81.02%  (0.7933- 0.8271) | 90.24%  (0.8897-0.9152) | 80.24%  (0.7852-0.8196) |
|  | PUSH | 81.16%  (0.7905-0.8237) | 91.34%  (0.8983-0.9286) | 77.54%  (0.7529- 0.7979) | 88.46%  (0.8674-0.9019) | 82.22%  (0.8016-0.8428) |
|  | Total | 80.30%  (0.7939-0.8121) | 90.21%  (0.8953-0.9089) | 78.27%  (0.7733- 0.7921) | 89.44%  (0.8874-0.9014) | 78.97%  (0.7804-0.7990) |
| Same stain & different scanners | Scanner 1  (40X) | 76.01%  (0.7227-0.7975) | 88.30%  (0.8548-0.9112) | 73.64%  (0.6978-0.7750) | 85.29%  (0.8218-0.8839) | 83.20%  (0.7992-0.8648) |
|  | Scanner 2  (40X) | 77.63%  (0.7398-0.8129) | 89.53%  (0.8685-0.9222) | 73.84%  (0.6998-0.7769) | 88.68%  (0.8591-0.9146) | 77.60%  (0.7395-0.8125) |
|  | Scanner 3  (20X) | 76.63%  (0.7292-0.8034) | 89.05%  (0.8631-0.9179) | 76.97%  (0.7328-0.8066) | 88.73%  (0.8596-0.9150) | 77.60%  (0.7395-0.8125) |
|  | Scanner 4  (40X) | 78.33%  (0.7471-0.8194) | 90.16%  (0.8755-0.9277) | 81.38%  (0.7797-0.8479) | 90.73%  (0.8819-0.9327) | 83.40%  (0.8014-0.8666) |
| Same scanner & different stains | BD Pap | 77.56%  (0.7390-0.8121) | 88.83%  (0.8607-0.9159) | 79.36%  (0.7581-0.8291) | 89.47%  (0.8678-0.9216) | 77.40%  (0.7373-0.8107) |
|  | TP Pap | 81.33%  (0.7792-0.8475) | 90.61%  (0.8805-0.9317) | 80.08%  (0.7657-0.8358) | 90.65%  (0.8810-0.9320) | 80.60%  (0.7713-0.8407) |
|  | BD HE | 81.08%  (0.7765-0.8451) | 91.42%  (0.8897-0.9388) | 82.28%  (0.7894-0.8563) | 91.64%  (0.8922-0.9407) | 83.40%  (0.8014-0.8666) |

SMUH: Southern Medical University's Nan Fang Hospital; ZUFAH: The Zhengzhou University First Affiliated Hospital; PUSH: Peking University Shenzhen Hospital. BD Pap: BD SurePath system with Papanicolaou stain; TP Pap: ThinPrep with Pap stain; BD HE: BD SurePath with Hematoxylin and Eosin stain.

**Table S9 The external validation and Image Appearance Migration (IAM).**

|  |  | Sensitivity  (95% CI) | Specificity  (95% CI) | PPV  (95% CI) | NPV  (95% CI) | Accuracy  (95% CI) |
| --- | --- | --- | --- | --- | --- | --- |
| FCH | AI | 79.54%  (0.7377-0.8531) | 93.10%  (0.8947-0.9672) | 79.50%  (0.7373-0.8527) | 93.24%  (0.8965-0.9683) | 79.79%  (0.7405-0.8553) |
|  | AI+IAM | 79.92%  (0.7377-0.8531) | 93.27%  (0.8947-0.9672) | 79.95%  (0.7373-0.8527) | 93.45%  (0.8965-0.9683) | 80.32%  (0.7405-0.8553) |
| WCH | AI | 78.97%  (0.7301-0.8492) | 93.33%  (0.8968-0.9697) | 80.58%  (0.7480-0.8636) | 93.60%  (0.9002-0.9718) | 82.22%  (0.7664-0.8781) |
|  | AI+IAM | 81.88%  (0.7626-0.8751) | 94.78%  (0.9153-0.9803) | 83.09%  (0.7761-0.8856) | 94.99%  (0.9180-0.9818) | 85.56%  (0.8042-0.9069) |
| ANHU | AI | 77.12%  (0.7144-0.8280) | 92.53%  (0.8897-0.9608) | 75.32%  (0.6949-0.8115) | 92.63%  (0.8910-0.9617) | 78.10%  (0.7250-0.8369) |
|  | AI+IAM | 81.89%  (0.7669-0.8710) | 93.82%  (0.9056-0.9707) | 79.37%  (0.7390-0.8484) | 93.93%  (0.9070-0.9716) | 81.90%  (0.7670-0.8711) |
| ZUFAH | AI | 86.10%  (0.8275-0.8944) | 93.65%  (0.9129-0.9601) | 83.08%  (0.7946-0.8671) | 92.75%  (0.9024-0.9525) | 81.27%  (0.7749-0.8504) |
|  | AI+IAM | 87.99%  (0.8485-0.9113) | 94.62%  (0.9244-0.9680) | 80.92%  (0.7712-0.8472) | 94.31%  (0.9207-0.9655) | 84.67%  (0.8119-0.8815) |
| PUSH | AI | 87.49%  (0.8436-0.9063) | 94.30%  (0.9211-0.9650) | 88.26%  (0.8521-0.9131) | 94.39%  (0.9221-0.9657) | 85.05%  (0.8167-0.8843) |
|  | AI+IAM | 87.03%  (0.8385-0.9022) | 94.48%  (0.9232-0.9664) | 88.36%  (0.8532-0.9140) | 95.11%  (0.9307-0.9715) | 85.98%  (0.8269-0.8927) |
| HHMG | AI | 81.64%  (0.7964-0.8364) | 93.80%  (0.9256-0.9505) | 82.09%  (0.8010-0.8407) | 93.77%  (0.9253-0.9502) | 81.99%  (0.8000-0.8398) |

AI: AI-TFNA; AI+IAM: IAM-enhanced model. FCH: Fujian Cancer Hospital; WCH: West China Hospital, Sichuan University; AHNU: Affiliated Hospital of Nantong University; ZUFAH: The Zhengzhou University First Affiliated Hospital; PUSH: Peking University Shenzhen Hospital; HHMG: Huayin Health Medical Group.

**Table S10 Image Appearance Migration pseudo code for different sample conditions**

| Algorithm 1: Image Appearance Migration (IAM) |
| --- |
| 1. **Input:** (1) Source dataset: different sample conditions; (2) Reference dataset: SMUH WSIs 2. **Output:** IAM results of WSI from unknown sample conditions 3. **Step l:** Tissue Mask Extraction;  - **Otsu's method:** If the input image is in color, convert it to a grayscale image to simplify the analysis, as Otsu's method operates on a single-channel image. - **Post-Processing:** Perform morphological operations (e.g., dilation. erosion) on the binary mask to remove noise and enhance the mask quality.  1. **Step 2:** Calculate the RGB mean of reference and source dataset;  - Calculate the RGB mean of the reference tissue area:  1. $\alpha=\left[ \frac{\sum_{i=1}^{N} meanR^{i}}{N},\frac{\sum_{j=1}^{N} meanG^{j}}{N},\frac{\sum_{k=1}^{N} meanB^{k}}{N} \right]$  - Calculate the RGB mean of the source tissue area:  1. $\beta=\left[ \frac{\sum_{i=1}^{M} meanR^{i}}{M},\frac{\sum_{j=1}^{M} meanG^{j}}{M},\frac{\sum_{k=1}^{M} meanB^{k}}{M} \right]$ 2. Among the formulations, ***N*** and ***M*** represent the total number of pixels in the reference tissue area and the source tissue area, respectively.  - Adjustment Factor:  1. $adjustment\_factor=\frac{\alpha}{\beta}$ 2. **Step 3:** Image Appearance Migration of Image ***I*** from source dataset; 3. $I_{IAM}=min(\left[ I_{R}(x,y)\cdot\frac{\boldsymbol{\alpha}}{\boldsymbol{\beta}}{,I}_{G}(x,y)\cdot\frac{\boldsymbol{\alpha}}{\boldsymbol{\beta}}{,I}_{B}(x,y)\cdot\frac{\boldsymbol{\alpha}}{\boldsymbol{\beta}} \right]$, 255) |

**REFERENCES:**

1. Zhang Y, Lu F, Shi H, Guo LH, Wei Q, Xu HX, Zhang YF. Predicting malignancy in thyroid nodules with benign cytology results: The role of Conventional Ultrasound, Shear Wave Elastography and BRAF V600E. *Clin Hemorheol Microcirc*. 2022;81:33-45. DOI: 10.3233/CH-211337.

2. Huang G, Liu W, Han L, Zhang Y, Liu S, Zhang J, Niu B. Age and BRAF(V600E) Mutation Stratified Patients with Cytologically Benign Thyroid Nodules. *Int J Gen Med*. 2023;16:6025-6039. DOI: 10.2147/IJGM.S443711.

3. Yücel Z, Akal F, Oltulu P. Mitotic cell detection in histopathological images of neuroendocrine tumors using improved YOLOv5 by transformer mechanism. *Signal, Image and Video Processing*. 2023;17:4107-4114.

4. Redmon J, Divvala S, Girshick R, Farhadi A, eds. You only look once: Unified, real-time object detection. In; 2016: 779-788.

5. Vahadane A, Peng T, Sethi A, Albarqouni S, Wang L, Baust M, Steiger K, Schlitter AM, Esposito I, Navab N. Structure-preserving color normalization and sparse stain separation for histological images. *IEEE Trans Med Imaging*. 2016;35:1962-1971.
